# Deep Unfolding of Iteratively Reweighted ADMM for Wireless RF Sensing

**DOI:** 10.3390/s22083065

**Published:** 2022-04-15

**Authors:** Udaya S. K. P. Miriya Thanthrige, Peter Jung, Aydin Sezgin

**Affiliations:** 1 Institute of Digital Communication Systems, Ruhr University Bochum, 44801 Bochum, Germany; aydin.sezgin@rub.de; 2 Institute of Communications and Information Theory, Technical University Berlin, 10587 Berlin, Germany; peter.jung@tu-berlin.de; 3 Data Science in Earth Observation, Technical University of Munich, 82024 Munich, Germany

**Keywords:** algorithm unfolding, clutter suppression, defects detection, compressive sensing, reweighted norm

## Abstract

We address the detection of material defects, which are inside a layered material structure using compressive sensing-based multiple-input and multiple-output (MIMO) wireless radar. Here, strong clutter due to the reflection of the layered structure’s surface often makes the detection of the defects challenging. Thus, sophisticated signal separation methods are required for improved defect detection. In many scenarios, the number of defects that we are interested in is limited, and the signaling response of the layered structure can be modeled as a low-rank structure. Therefore, we propose joint rank and sparsity minimization for defect detection. In particular, we propose a non-convex approach based on the iteratively reweighted nuclear and $\ell_{1}$-norm (a double-reweighted approach) to obtain a higher accuracy compared to the conventional nuclear norm and $\ell_{1}$-norm minimization. To this end, an iterative algorithm is designed to estimate the low-rank and sparse contributions. Further, we propose deep learning-based parameter tuning of the algorithm (i.e., algorithm unfolding) to improve the accuracy and the speed of convergence of the algorithm. Our numerical results show that the proposed approach outperforms the conventional approaches in terms of mean squared errors of the recovered low-rank and sparse components and the speed of convergence.

## 1. Introduction

The electromagnetic (EM) waves-based remote sensing has many potential applications such as behind the wall object identification [1], multi-layer target detection [2], material characterization [3], defect detection [4,5,6,7], and many more. In EM and radio frequency (RF) waves-based detection of objects/defects which are behind or inside a layered structure, the EM waves that reflect from the object/defect are analyzed. Here, one major challenge is the presence of strong unwanted reflections, i.e., clutter [1,8]. In this context, the main source of the clutter is the reflection from the surface of the layered material structure.

The state-of-the-art clutter suppression methods such as background subtraction (BS), time-gating, and subspace projection (SP) [9] are not able to suppress the clutter in the context of object/defect detection. This is due to the fact that in BS, it requires the reference data of the scene, and this reference data is not available most of the time. Moreover, in the SP, prior knowledge is required to determine the perfect threshold for clutter removal. On the other hand, in time-gating, the time window in which clutter resides needs to be determined for successful clutter removal. However, this time window cannot be determined exactly. Clutter suppression becomes even more challenging if objects and clutter are closely located. This occurs regularly in the detection of defects which are inside a layered structure. Then, due to the small delay spread, the signaling responses of defects and clutter superimpose each other. In order to overcome these challenges, advanced signal processing methods are required for clutter suppression [1,8,10].

In many scenarios, the responses of the material defects are weak and, thus, difficult to detect. Even if there is no clutter, due to very low signal amplitude, it may be difficult to detect material defects in the presence of noise. In this context, the weak signal detection in the presence of noise has drawn attention in the defect detection research field. Therefore, we briefly discuss the weak signal detection in the following. Stochastic resonance has been widely used in weak signal detection [11,12,13]. In [11], to improve upon the weak signal detection by stochastic resonance, the relationship between the current and the previous value of the state variable of the system has been utilized. It is worth noticing that weak signal detection plays an important role in other applications such as health monitoring. Similar to defect detection, health monitoring aims to detect weak signals in the presence of strong noise. In [14], a comparative study of well-known adaptive mode decomposition approaches that are used for the aforementioned task is reviewed. Here, the advantages, limitations, and the performance comparison of adaptive mode decomposition approaches, namely empirical mode decomposition, Hilbert vibration decomposition, and variational mode decomposition, have been given. Other than signal detection, the extraction of features of the detected signal is important in many applications as these features are used for classification and clustering. In this context, it is important to select the most important features as the accuracy and speed of the classification depend on the features that are used. The impact of the feature selection for electromyographic signal decomposition is studied in [15]. Moreover, in this study, various feature extraction methods are compared, and a guide to select the most important features that improve the signal decomposition is provided [15]. As we discussed above, weak signal detection in the presence of disturbances like noise or clutter is challenging, therefore, advanced signal procession methods are required. Next, we discuss clutter suppression in more detail.

In many scenarios, the number of defects is limited. Therefore, the signaling response of the defect is sparse in nature. By exploring this, compressive sensing (CS) [16] based approaches have shown promising results in object/defect detection with clutter [1,8]. In addition, the CS-based approaches do not require a full measurement data set, which results in fast data acquisition and less sensitivity to sensor failure, wireless interference, and jamming. In CS-based approaches, it is considered that the clutter resides in a low-rank subspace and the response of the objects is sparse [1,8].

To this end, we present a general data acquisition model where the received data vector $y\in C^{K}$ is modeled as a combination of a low-rank matrix $L\in C^{M\times N}$ and a sparse matrix $S\in C^{M\times N}$ with M≤N:

(1) $$y=A_{l}\mathrm{vec}\left(L\right)+A_{s}\mathrm{vec}\left(S\right)+n,$$

in which $A_{l},\;A_{s},\in C^{K\times MN}$ with K≪MN, $n\;\in C^{K}$ are compression operators/measurement matrices and measurement noise, respectively. Here, the compression ratio is defined as K/MN. Further, vec(·) denotes the vectorization operator, which converts a matrix to a vector by stacking the columns of the matrix. Given the received data vector y, our aim is to estimate the signals of interest, LandS, using a small number of linear measurements by minimizing the rank and sparsity as

(2) $$\begin{array}{cc} \left\{\hat{L},\hat{S}\right\} & =\underset{L,\;S}{arg min}\lambda_{l}\;\mathrm{rank}\left(L\right)+\lambda_{s}{∥S∥}_{0},\\ \; & \;\;\;s.t.\;{∥y-A_{s}\mathrm{vec}\left(S\right)-A_{l}\mathrm{vec}\left(L\right)∥}_{2}^{2}\;\leq \;\epsilon , \end{array}$$

where $\lambda_{l}$, $\lambda_{s}$ are regularization parameters and ϵ is a small positive constant (noise bound). Here, ${∥\cdot ∥}_{0}$ is the $\ell_{0}$-norm, i.e., sparsity (the number of non-zero components). Note that the problem given in (2) is also known as robust principal component analysis (RPCA) [17]. The RPCA problem has different types as follows: (a) standard/classical RPCA in which both $A_{l}$ and $A_{s}$ in (1) are identity matrices [17], (b) the matrices $A_{l}=A_{s}=A$ and A is a selection operator which select a random subset of size *K* from MN entries [18], (c) both $A_{l}$ and $A_{s}$ are K×MN matrices which map the vector space $C^{MN}$ to the vector space $C^{K}$ [18].

The problem given in (2) is an NP-hard problem and, thus, difficult to solve. To this end, convex relaxations of sparsity and rank in terms of $\ell_{1}$-norm of a matrix (absolute sum of elements) and nuclear norm of a matrix (sum of singular values) are utilized, respectively [19,20,21]. However, enjoying a rigorous analysis, the convex relaxations of sparsity and rank cause disadvantages in many applications. In addition to that, in many applications, the important properties of the signal are preserved by the large coefficients/singular values of the signal [22]. However, the $\ell_{1}$-norm/nuclear norm minimization algorithms shrink all the coefficients/singular values with the same threshold. Thus, to avoid this weakness, we should shrink less the larger coefficients/singular values. To address aforementioned drawbacks, non-convex approaches such as reweighted nuclear norm and reweighted $\ell_{1}$-norm minimization have been considered [22,23,24,25]. These non-convex approaches have shown better performance over the convex relaxations by providing tighter characterizations of rank and sparsity, yet their behavior and convergence have not been fully studied [26].

Generally, RPCA problems are numerically solved by means of iterative algorithms based on the alternating direction method of multipliers (ADMM) [17,27,28] or accelerated proximal gradient (APG) [29,30]. In iterative algorithms, the accuracy of the recovered signal component and the convergence rate depends on the proper selection of parameters (e.g., regularization/thresholding/denoising parameters). Generally, parameters are chosen by handcrafting, and it is a time-consuming task. In this context, machine learning-based parameter tuning using training data has shown promising results in many applications such as sparse vector recovery [31,32,33] and image processing [34]. For instance, as shown in [31], the unfolded iterative soft-thresholding algorithm (LISTA) converges twenty times faster than the conventional iterative soft-thresholding algorithm (ISTA). This approach is known as algorithm unrolling/unfolding, and an overview can be found in [35].

In this work, we formulate the detection of material defects as a RPCA problem. This RPCA problem is solved based on the reweighted nuclear norm and reweighted $\ell_{1}$-norm minimization. However, most of the time, RPCA problems are solved by using the convex relaxation or with the single reweighting, i.e., either reweighted $\ell_{1}$-norm or reweighted nuclear norm [22,30,36,37]. Next, our objective is to jointly estimate the low-rank matrix and the sparse matrix from few compressive measurements. It is worth noticing that most of the work in the literature focuses on the standard RPCA problem, where $A_{l}$ and $A_{s}$ are identity matrices [22,36]. To the best of our knowledge, the full doubly reweighted (joint reweighted nuclear norm and reweighted $\ell_{1}$-norm) approach has not yet been studied comprehensively in the literature for the compressive case. Then, we propose an iterative algorithm for (locally) minimizing the objective, i.e., reweighted nuclear norm and reweighted $\ell_{1}$-norm, which is based on the alternating direction method of multipliers (ADMM) [38,39]. Further, we propose deep learning-based parameter tuning to improve the accuracies of the recovered low-rank and sparse components and the convergence rate of the ADMM-based iterative algorithm.

In addition to the EM-based defect detection, there are many applications where the data generated by the application can be modeled as a combination of low-rank plus sparse contributions. For instance, in video surveillance, the static background results in a low-rank contribution, and moving objects result in a sparse contribution [40]. Further, in human face recognition from a corrupted face image, the human face can be approximated as a low-rank structure while self-shadowing and specularities are modeled as sparse contributions [40,41]. Therefore, RPCA can be applied to the aforementioned applications and other applications as long as the data/measurements are combinations of low-rank and sparse contributions. It is worth noticing that our proposed full doubly reweighted (joint reweighted nuclear norm and reweighted $\ell_{1}$-norm) approach with deep learning-based parameter tuning for RPCA is not limited to EM-based defect detection and can be applied to other applications that are solved using RPCA.

In the context of the algorithm unfolding for the RPCA, the convolutional robust principal component analysis (CORONA) [30,37] are the closest studies to our work. There are fundamental methodological differences between our work and [30,37]: (a) Both [30,37] considered the standard convex relaxation ($\ell_{1,2}$-norm and nuclear norm) to solve the RPCA problem, while we propose the reweighted $\ell_{1}$-norm and reweighted nuclear norm. (b) In this work, the RPCA problem is solved by an iterative algorithm based on ADMM, while the iterative algorithm in [30,37] is based on fast ISTA (FISTA). The motivation to propose ADMM over ISTA/FISTA for RPCA is as follows. As shown in [17,27] for RPCA, the ADMM-based approach is able to achieve the desired solution with a good recovery error with few iterations for a wide range of applications compared to APG-based approaches like ISTA/FISTA. Further, the performances of the APG-based approaches are heavily dependent on the good continuation schemes [17]. This condition may not be satisfied for a wide range of applications. (c) Different from [30,37], our focus is on defect detection based on the stepped-frequency continuous wave (SFCW) radar, while [30,37] focus on ultrasound imaging application. Moreover, experimental measurement data of [30,37] have considered that $A_{l}=A_{s}=A$ in (1) is an identity matrix, while we consider both scenarios where A is an identity matrix and it is a compression operator. Further, for the SFCW radar application, we consider that $A_{l}\neq A_{s}$. Further, we have studied the performance of our approach with a generic real-valued Gaussian model for different compression ratios.

The CORONA focuses on ultrasound imaging applications where sparse matrix has row-sparse structure. Thus, there is a strong relationship between measurement to measurement, and there is a common sparsity structure. Therefore, $\ell_{1,2}$-norm minimization is more suitable than $\ell_{1}$-norm minimization to estimate sparse matrix S. Further, the CORONA is based on a convolutional deep neural network to learn spatial invariance features of data, which is more suitable for ultrasound imaging applications than a dense deep neural network (DNN). However, we assume that there is no strong relationship of a data element to its neighboring elements, nor is there a specific sparsity structure. Thus, we consider a dense DNN in this work. It is straightforward to modify our ADMM approach with convolutional DNN and the $\ell_{1,2}$-norm minimization. In CORONA [30], customized complex-valued convolution layers and singular value decomposition operations are utilized. In our work, we have implemented a dense DNN which supports complex-valued data and singular value decomposition (SVD) operation. The contributions of this work are summarized as follows:

### 1.1. Contribution

- We propose a generic approach based on the non-convex fully double-reweighted approach, i.e., both reweighted $\ell_{1}$-norm and reweighted nuclear norm simultaneously to solve the RPCA problem. To this end, we propose an iterative algorithm based on ADMM to estimate the low-rank and sparse components jointly.
- In contrast to standard/classical RPCA, we consider the compressive sensing data acquisition model, which reflects more on the practical problem at hand. Next, to improve the accuracy and convergence speed of the ADMM-based iterative algorithm, we propose a deep neural network (DNN) to tune the parameters of the iterative algorithm (i.e., algorithm unfolding/unrolling) from training data.
- We intensively evaluate our proposed approach for a generic Gaussian data acquisition model with $A_{l}=A_{s}=A$. In addition to that, the defect detection by SFCW radar from compressive measurements with $A_{l}\neq A_{s}$ is considered. To compare our approach, we consider the standard convex approach (i.e., nuclear norm and $\ell_{1}$-norm minimization) and the untrained ADMM-based iterative algorithm for different compression ratios. In both the generic Gaussian data acquisition model and SFCW-based defect detection, our numerical results show that the proposed approach outperforms the conventional approaches in terms of mean squared errors of the recovered low-rank and sparse components and the speed of convergence.
- In the context of algorithm unrolling for RPCA, we compare our approach with the approach given in [30] (CORONA). It turns out that our proposed approach shows similar performance as CORONA for experimental ultrasound imaging data used in [30], and our approach outperforms CORONA for generic Gaussian data. It is worth noticing that there is a row-sparse nature of the experimental ultrasound data. That is the reason CORONA uses $\ell_{1,2}$-norm minimization to estimate sparse matrix S. Our approach is generic, yet our approach is able to achieve similar results as CORONA by learning. This shows the applicability of our approach to different types of use cases and data (defect detection, ultrasound imaging, generic Gaussian data).
- We numerically analyze the robustness of our proposed approach for the generic Gaussian data acquisition model. Here, we consider the deviation in the measurement matrices ($A_{l},\;A_{s}$) and testing signal-to-noise ratio (SNR) uncertainty. It was observed that the proposed approach is robust for a small deviation in the measurement matrices. Further, it was observed that training with the SNR like 5 dB is favorable when SNR of the testing data is unknown.

The remainder of the paper is organized as follows. We introduce the SFCW radar-based defect detection and the low-rank plus sparse recovery with reweighting in Section 2. In Section 3, we discuss the DNN-based low-rank plus sparse recovery algorithm unfolding. In Section 4, we provide an evaluation of the proposed DNN-based low-rank plus sparse recovery algorithm unfolding approaches and provide interesting insights. Section 5 concludes the paper.

### 1.2. Notation

In this paper, the following notation is used. A vector is denoted in boldface lower-case letter, while the matrices are denoted in boldface upper-case. The $\ell_{0}$-norm (the number of nonzero components), $\ell_{1}$-norm (absolute sum of elements) of a matrix/vector, and nuclear norm of a matrix (sum of singular values) are denoted by ${∥\cdot ∥}_{0}$, ${∥\cdot ∥}_{1}$, and ${∥\cdot ∥}_{*}$, respectively. Further, the Frobenius norm of a matrix and $\ell_{2}$-norm is given by ${∥\cdot ∥}_{F}$ and ${∥\cdot ∥}_{2}$, respectively. The Hermitian and transpose of the matrix A are represented by $A^{H}$ and $A^{T}$, respectively. In addition, the Moore–Penrose pseudo inverse is denoted by ${\left(\cdot \right)}^{†}$. A matrix of size M×N with all elements equal to zero and one are denoted by $0_{M,N}$ and $1_{M,N}$, respectively. Moreover, a vector of size *M* with all elements equal to zero and one are denoted by $0_{M}$ and $1_{M}$, respectively. In addition, identity matrix is denoted by I. The main variable list and abbreviations that are used in this manuscript are listed at the end of the manuscript.

## 2. System Model

First, we briefly present the system model of the mono-static SFCW radar-based defect detection. Next, we discuss the ADMM -based iterative algorithm for the low-rank plus sparse recovery.

### 2.1. SFCW Radar Based Defect Detection

**Figure 1:**
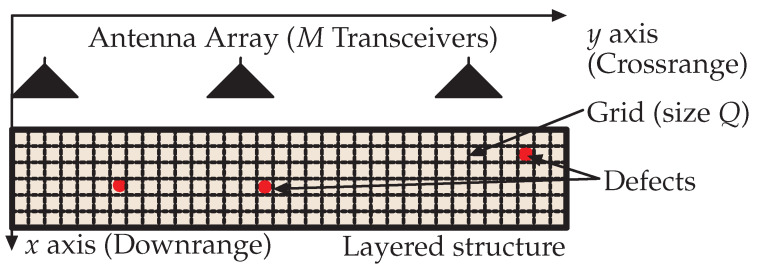
Getting the measurements of a single-layered material structure using an SFCW radar with *M* transceivers. The received signal consists of two main components, the reflection of the layered material structure ($Y^{l}$) and the reflection of the defects ($Y^{d}$), where $Y^{l}$ is the main clutter source. Here, defects are shown as red circles.

We consider an SFCW radar with *M* transceivers which are placed in parallel to the single-layered material structure while maintaining an equal distance between transceivers, as shown in Figure 1. In SFCW radar, each transceiver transmits a stepped-frequency signal containing *N* frequencies which are equally spaced over the bandwidth of *B* Hz. To this end, the received signal corresponding to all *M* transceivers and *N* frequencies $Y\in C^{M\times N}$ are given by

(3) $$Y=Y^{l}+Y^{d}+Z.$$

Note that Y consists of two main components, the reflection of the layered material structure ($Y^{l}$) and the reflection of the defects ($Y^{d}$). Here, Z is the additive Gaussian noise matrix. Next, we discuss in detail the modeling of the received signal of the defects by using the propagation time delay. To this end, the scene shown in Figure 1 is virtually partitioned into a rectangular grid of size *Q*. Suppose that the round-travel time of the signal from the *m*-th antenna location to the *p*-th defect and back is given by $\tau_{m,p}$. Then, the received signal of the defects $y_{m,n}^{d}$ in *m*-th transceiver corresponding to *n*-th frequency band $f_{n}$ is given by [1]

(4) $$y_{m,n}^{d}=\sum_{p=1}^{P}\alpha_{p}\;\exp\left(-j2\pi f_{n}\tau_{m,p}\right).$$

Here, $j\;=\;\sqrt{-1}$, $\alpha_{p}\in C$ is the complex reflectivity coefficient of the *p*-th defect, and *P* is the total number of defects. To this end, $\mathrm{vec}\left(Y^{d}\right)\in C^{MN\times 1}$ is given by

(5) $$\mathrm{vec}(Y^{d})=Ds,$$

where $s\in C^{Q\times 1}$ contains all the $\alpha_{p}$ values of the defects. Since there are *P* defects, the vector s only contains *P* non-zero entries. The matrix D is given by ${\left[{\left(D_{1}\right)}^{T},\ldots ,{\left(D_{m}\right)}^{T},\ldots ,{\left(D_{M}\right)}^{T}\right]}^{T}\in C^{MN\times Q}$. Note that the (n,q)-th element of the matrix $D_{m}\in C^{N\times Q}$ is given by $\exp(-j2\pi f_{n}\tau_{m,q})$, where $\tau_{m,q}$ is the propagation time delay between the *m*-th antenna to the *q*-th grid location. We assume that the propagation time delays $\tau_{m,p}$ of the defects are exactly matched with the propagation time delays of the grid locations. If this condition does not satisfy, it is known as grid mismatch. The grid mismatch degrades the performance of the sparse signal estimation [42]. There are several approaches proposed to rectify this problem, e.g., Bayesian learning-based approach [43], iterative dictionary updates [3], and many more. Similar to the received signal of the defects $y_{m,n}^{d}$, the received signal of the layered material structure $y_{m,n}^{l}$ in *m*-th transceiver corresponding to *n*-th frequency band $f_{n}$ is given by [1]:

(6) $$y_{m,n}^{l}=\sum_{\bar{p}=1}^{\bar{P}+1}\alpha_{l}a_{\bar{p}}\;\exp\left(-j2\pi f_{n}\tau_{m,\bar{p}}\right).$$

Here, $\alpha_{l}\in C$ is the complex reflectivity of the layered material structure. $a_{\bar{p}}$ and $\tau_{m,\bar{p}}$ are the propagation loss and the propagation delay of the $\bar{p}$-th return of the layered material structure. The number of internal reflections within the layered material is given by $\bar{P}$.

### 2.2. Compressed Sensing (CS) Approach

In the compressed sensing (CS) setup, only a subset of antennas/frequencies are available or selected. Now, the reduced data vector $y_{cs}\in C^{K\times 1}$ of size K(≪MN) is given by

(7) $$\begin{array}{c} y_{cs}=\Phi \left(\mathrm{vec}(Y)\right)=\Phi \mathrm{vec}\left(Y^{l}\right)+\Phi Ds+\Phi \mathrm{vec}\left(Z\right), \end{array}$$

where $\Phi \in R^{K\times MN}$ is the selection matrix. The matrix Φ has a single non-zero element of value one in each row to indicate the selected frequency of a particular antenna if that antenna is selected. Here, our main objective is to recover $Y^{l}$ and s from the reduced data vector $y_{cs}$ using the low-rank plus sparse recovery approach as detailed below.

### 2.3. Low-Rank Plus Sparse Recovery Algorithm

From now on we consider the general data acquisition model given in (1) in Section 1, i.e., $y=A_{l}\mathrm{vec}\left(L\right)+A_{s}\mathrm{vec}\left(S\right)+n$. Note that the SFCW radar model given in (7) is mapped to the generic measurement model by considering $A_{s}\;=\;\Phi D$, $A_{l}\;=\;\Phi$, $Y^{l}\;=\;L$, s=vec(S), and $y_{cs}\;=\;y$, respectively. Our objective is to recover the low-rank matrix L and the sparse matrix S from the compressive measurements y. Thus, the estimation of L and S from y is done by minimizing rank and the sparsity ($\ell_{0}$-norm). Note that rank and $\ell_{0}$-norm minimization problems are usually NP-hard. Thus, one may use instead convex relaxations based on the nuclear norm of a matrix and $\ell_{1}$-norm of a matrix as follows:

(8) $$\begin{array}{cc} \left\{\hat{L},\hat{S}\right\} & =\underset{L,\;S}{arg min}\lambda_{l}{∥L∥}_{*}+\lambda_{s}{∥S∥}_{1},\\ \; & \;\;\;s.t.\;{∥y-A_{s}\mathrm{vec}\left(S\right)-A_{l}\mathrm{vec}\left(L\right)∥}_{2}^{2}\;\leq \;\epsilon . \end{array}$$

The resulting convex problems, i.e., $\ell_{1}$-norm and nuclear norm minimization, are well studied in the literature, and there are several non-convex approaches to improve over the standard convex relaxation. One well-known approach is iterative reweighting of the $\ell_{1}$-norm [23,32,44] and nuclear norm [22,45,46,47]. Alternating direction method of multipliers (ADMM) is used to solve the problem given in (8). First, we formulate the problem given in (8) based on ADMM approach, and then we introduce the non-convex double-reweighted approach, i.e., both reweighted $\ell_{1}$-norm and reweighted nuclear norm simultaneously. Let the signal component value of S and L at the *t*-th iteration be denoted as ${\left(\cdot \right)}^{t}$. Now, based on the ADMM, S and L are estimated by

(9) $$\begin{array}{cc} L^{t+1} & =\underset{L}{arg min}\;\lambda_{l}{∥L∥}_{☆}+\frac{\rho }{2}{∥A_{s}\mathrm{vec}\left(S^{t}\right)+A_{l}\mathrm{vec}\left(L\right)-y+\frac{1}{\rho }u^{t}∥}_{2}^{2}, \end{array}$$

(10) $$\begin{array}{cc} S^{t+1} & =\underset{S}{arg min}\;\lambda_{s}{∥S∥}_{1}+\frac{\rho }{2}{∥A_{s}\mathrm{vec}\left(S\right)+A_{l}\mathrm{vec}\left(L^{t+1}\right)-y+\frac{1}{\rho }u^{t}∥}_{2}^{2}, \end{array}$$

(11) $$u^{t+1}=u^{t}+\rho \left(A_{s}\mathrm{vec}\left(S^{t+1}\right)+A_{l}\mathrm{vec}\left(L^{t+1}\right)-y\right).$$

Here, u, ρ>0 are auxiliary variables and a penalty factor. Let $\sigma \left(L\right)\;=\;\left[\sigma_{1},\ldots \sigma_{m},\ldots ,\sigma_{M}\right]\in \;R^{M}$ be the singular values of L. The nuclear norm of L is given by ${∥L∥}_{☆}={∥\sigma (L)∥}_{1}$. Now, we are going to introduce the weighted $\ell_{1}$-norm and weighted nuclear norm to the sub-problems given in (9) and (10) as follows:

(12) $$\begin{array}{cc} L^{t+1} & =\underset{L}{arg min}\;\lambda_{l}{∥w_{l}^{t}⊙\sigma \left(L\right)∥}_{1}+\frac{\rho }{2}{∥A_{s}\mathrm{vec}\left(S^{t}\right)+A_{l}\mathrm{vec}\left(L\right)-y+\frac{1}{\rho }u^{t}∥}_{2}^{2}, \end{array}$$

(13) $$\begin{array}{cc} S^{t+1} & =\underset{S}{arg min}\;\lambda_{s}{∥w_{s}^{t}⊙S∥}_{1}+\frac{\rho }{2}{∥A_{s}\mathrm{vec}\left(S\right)+A_{l}\mathrm{vec}\left(L^{t+1}\right)-y+\frac{1}{\rho }u^{t}∥}_{2}^{2}. \end{array}$$

The operator ⊙ denotes element-wise multiplication. Here, $w_{l}^{t}\;\in \;R^{M}$ and $w_{s}^{t}\;\in \;R^{MN}$ are non-negative weight vectors in t+1-th iteration. To this end, $w_{l}^{t}$ and $w_{s}^{t}$ are calculated based on the previous estimation of the L and S, i.e., $L^{t}$ and $S^{t}$.

(14) $$w_{l}^{t}=g_{l}\left(\sigma \left(L^{t}\right)\right)\;\mathrm{and}\;w_{s}^{t}=g_{s}\left(|S^{t}|\right).$$

Here, $g_{l}\left(\cdot \right)$ and $g_{s}\left(\cdot \right)$ are decay functions, applied component-wise, which are used to calculate the weights. There are several decay functions proposed in the literature, and an overview of the nuclear norm is given in [47]. In this work, motivated by [32], we consider element-wise (adaptive) soft-thresholding as the proximal operator of the weighted $\ell_{1}$-norm. In addition, inspired by [48], element-wise (adaptive) singular value soft-thresholding (i.e., element-wise soft-thresholding on the singular values of a matrix) is used as a proximal operator of the weighted nuclear norm. Now, $L^{t+1}$ and $S^{t+1}$ are given by

(15) $$L^{t+1}={\mathrm{SVT}}_{\lambda_{LT}^{t}}\left(A_{l}^{*}\left(y-A_{s}\mathrm{vec}\left(S^{t}\right)+\frac{u^{t}}{\rho }\right)\right),$$

(16) $$S^{t+1}={\mathrm{ST}}_{\lambda_{ST}^{t}}\left(A_{s}^{*}\left(y-A_{l}\mathrm{vec}\left(L^{t+1}\right)+\frac{u^{t}}{\rho }\right)\right),$$

where SVT(·) and ST(·) are the element-wise singular value soft-thresholding and element-wise soft-thresholding operators [32,48], respectively. Note that ${\left(\cdot \right)}^{*}$ is a linear operator which back projects the vector into the target matrix subspace. There are two options for ${\left(\cdot \right)}^{*}$: (a) Hermitian transpose ${\left(\cdot \right)}^{H}$, as done in [32], or (b) Moore–Penrose pseudo inverse ${\left(\cdot \right)}^{†}$, as done in [1]. Next, we are going to discuss the element-wise (adaptive) soft-thresholding and the element-wise (adaptive) singular value soft-thresholding.

### 2.4. Element-Wise Soft-Thresholding and Singular Value Soft-Thresholding

In (16), $\lambda_{ST}^{t}=\left[\lambda_{ST}^{1,1},\ldots ,\lambda_{ST}^{m,n},\ldots ,\lambda_{ST}^{M,N}\right]$ contains the element-wise thresholds for S for the t+1-th iteration. These thresholds are derived based on the previous estimate S, i.e., $S^{t}$,

(17) $$\begin{array}{c} \lambda_{ST}^{m,n}=\lambda_{S}\;g_{s}\left(\right|s_{m,n}^{t}\left|\right). \end{array}$$

Here, $\lambda_{S}$ is a positive constant (soft-thresholding parameter), and $s_{m,n}^{t}$ is the m-th row and n-th column element of the *t*-th estimation of S, i.e., $S^{t}$. The same concept is also applied to the singular value soft-thresholding which is used in (15), as discussed next. In this work, we consider the same decay function for both sparsity and rank, i.e., $g_{s}\left(\cdot \right)=g_{l}\left(\cdot \right)$. In (15), $\lambda_{LT}^{t}=\left[\lambda_{LT}^{1},\ldots ,\lambda_{LT}^{m},\ldots \lambda_{LT}^{M}\right]$ contains the different thresholds calculated from the singular values of the previous estimate of L as given below:

(18) $$\begin{array}{c} \lambda_{LT}^{m}=\lambda_{L}\;g_{l}\left(\sigma_{m}^{t}\right). \end{array}$$

Here, $\sigma_{m}^{t}$ is the m-th singular value of $L^{t}$, and $\lambda_{L}$ is a positive constant (singular-value-soft-thresholding parameter). For completeness, definitions of the element-wise soft-thresholding and singular value soft-thresholding are given in Appendix A.1. Our objective is to tune the parameters $\lambda_{S}$ in (17) and $\lambda_{L}$ in (18) by using a deep neural network, as discussed next.

## 3. Unfolding ADMM-Based Low-Rank Plus Sparse Recovery Algorithm

**Figure 2:**
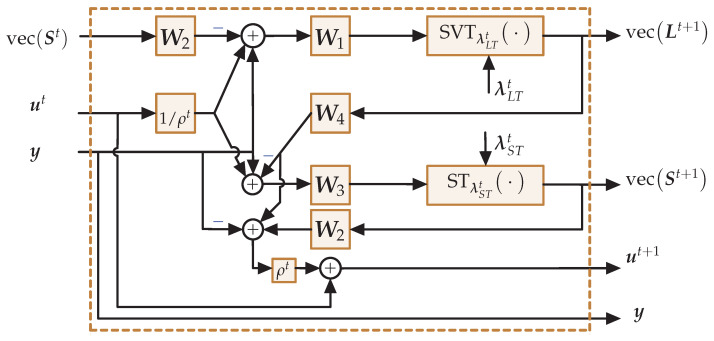
Block diagram of the *t*-th layer of the DNN which mimics the low-rank plus sparse recovery Algorithm 1. Weights of the linear layers ($W_{1},W_{2},W_{3},W_{4}$) and other parameters ($\lambda_{T}^{t}$, $\lambda_{S}^{t}$, $\gamma^{t}$, $\rho^{t}$) are learned from training data.

In this section, we are going to discuss the ADMM algorithm unfolding using a dense DNN. The iterative algorithm given in Algorithm 1 utilizes the ADMM steps given in (15), (16), and (11), and previous estimates are used in the next iteration. Thus, this kind of iterative algorithm can be considered as a recurrent neural network. The *t*-th iteration of the iterative Algorithm 1 is modeled as the *t*-th layer of the deep neural network as shown in Figure 2. Each matrix multiplication given in the ADMM steps (15), (16), and (11) are implemented as linear layers without biases. Here, our main objective is to learn the per iteration weights of the network and thresholding parameters $\lambda_{S}$ and $\lambda_{L}$ given in (17) and (18) from training data. To this end, the *t*-th layer of the neural network is represented by the following equations:

(19) $$L^{t+1}={\mathrm{SVT}}_{\lambda_{LT}^{t}}\left(W_{1}^{t}\left(y-W_{2}^{t}\mathrm{vec}\left(S^{t}\right)+\frac{u^{t}}{\rho^{t}}\;\right)\right),$$

(20) $$S^{t+1}={\mathrm{ST}}_{\lambda_{ST}^{t}}\left(W_{3}^{t}\left(y-W_{4}^{t}\mathrm{vec}\left(L^{t+1}\right)+\frac{u^{t}}{\rho^{t}}\right)\right),$$

(21) $$\begin{array}{c} u^{t+1}=u^{t}+\rho^{t}\left(W_{2}^{t}\mathrm{vec}\left(S^{t+1}\right)+W_{4}^{t}\mathrm{vec}\left(L^{t+1}\right)-y\right). \end{array}$$

Here, $W_{1}^{t}$, $W_{2}^{t}$, $W_{3}^{t}$, and $W_{4}^{t}$ are the weights of the *t*-th layer as shown in Figure 2. Their initial values are $W_{1}^{t}\;=\;A_{l}^{*}$, $W_{2}^{t}\;=\;A_{s}$, $W_{3}^{t}\;=\;A_{s}^{*}$, and $W_{4}^{t}\;=\;A_{l}$ to mimic the ADMM Algorithm 1. Further, $\lambda_{LT}^{t}$ and $\lambda_{ST}^{t}$ are the thresholding vectors of the *t*-th layer as given in (15) and (16). Note that $\lambda_{LT}^{t}$, and $\lambda_{ST}^{t}$ depend on the previous estimates of the $L^{t}$, $S^{t}$ and two parameters ($\lambda_{S}$ and $\lambda_{L}$). Here, we consider the weights $W_{1}^{t}$, $W_{2}^{t}$, $W_{3}^{t}$, and $W_{4}^{t}$ are tied over all the layers, i.e., sharing weights. However, we do not consider thresholding parameters ($\lambda_{S}$ and $\lambda_{L}$) γ and ρ to be tied over all layers, i.e., each layer has its own thresholding parameters. To this end, $\Theta =\left\{\lambda_{S}^{t},\lambda_{L}^{t},\gamma^{t},\rho^{t},W_{1},W_{2},W_{3},W_{4}\right\}$ represents the set of learning parameters. Here, $\lambda_{S}^{t}$ and $\lambda_{L}^{t}$ are the thresholding parameters of the *t*-th layer.
 **Algorithm 1:** Low-rank plus sparse recovery algorithm. 
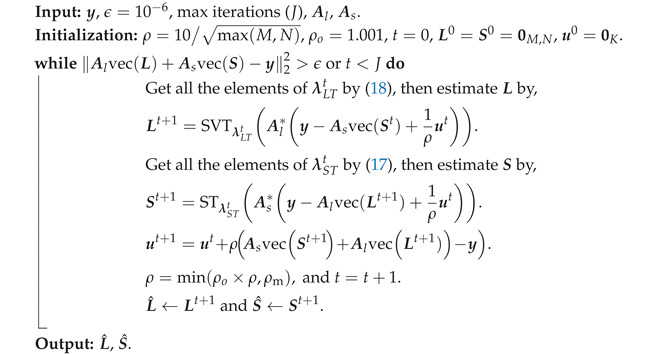


### 3.1. Training Phase

In the training phase, the DNN is trained in a supervised manner. Here, the DNN learns the parameters given in $\Theta =\left\{\lambda_{S}^{t},\lambda_{L}^{t},\gamma^{t},\rho^{t},W_{1},W_{2},W_{3},W_{4}\right\}$. Suppose that the DNN has *T* layers, then the outputs of the DNN in the training phase for the *i*-th sample are given by ${\hat{L}}_{i}$ and ${\hat{S}}_{i}$, respectively. Note that, in the training phase, the DNN minimizes the normalized mean squared error

(22) $$\mathrm{NMSE}=\frac{1}{T_{s}}\sum_{i=1}^{T_{s}}\left(\frac{1}{2}\frac{{∥{\hat{L}}_{i}-L_{i}∥}_{F}^{2}}{{∥L_{i}∥}_{F}^{2}}+\frac{1}{2}\;\frac{{∥{\hat{S}}_{i}-S_{i}∥}_{F}^{2}}{{∥S_{i}∥}_{F}^{2}}\right),$$

where $S_{i}$ and $L_{i}$ are *i*-th ground-truth low-rank and sparse matrices, and $T_{s}$ is the number of training samples.

In this work, in the context of DNN-based parameter tuning, we consider three versions of the ADMM-based iterative algorithm to solve the RPCA problem as follows: (a) Parameter tuning with non-adaptive thresholding (i.e., $g_{s}\left(x\right)=g_{l}\left(x\right)=1$). This approach is named as ADMM-based trained RPCA with thresholding (TRPCA-T). For the parameter tuning with adaptive thresholding, we consider two versions based on two decay functions as described in Section 2.4. These two approaches are named as follows: (b) ADMM-based trained RPCA with adaptive thresholding based on logarithm heuristic (TRPCA-AT(log)). (c) ADMM-based trained RPCA with adaptive thresholding based on exponential heuristic (TRPCA-AT(exp). Among above versions, in this work, we propose parameter tuning with adaptive thresholding approaches TRPCA-AT(log) and TRPCA-AT(exp) to solve the RPCA problem with the compressive sensing data acquisition model.

To have a comparison with our proposed approaches, we consider two approaches. In the first approach, we consider the untrained ADMM approach to solve the convex low-rank plus sparse recovery as given in Algorithm 1 with non-adaptive thresholding (i.e., $g_{s}\left(x\right)=g_{l}\left(x\right)=1$). This method is named as ADMM-based untrained RPCA with thresholding (URPCA-T). As a second option, we consider the low-rank plus sparse recovery problem given in (8). This method is named as low-rank plus sparse recovery with convex relaxation (LRPSRC).

### 3.2. Computation Complexity

In this subsection, the computational complexity of the proposed DNN is briefly discussed. Detail breakdown is given in Appendix A.2. The training complexity of the DNN is the addition of the feed-forward and the back propagation complexities. For $T_{s}$, number of training samples, $E_{p}$, number of epochs, and for M=N, the training computational complexity for the DNN with *T* layers is given by $O(TT_{s}E_{p}\left(N^{2}K+N^{3}\right))$. The testing computational complexity is the feed-forward propagation complexity of data through the DNN. It is given by $O(TN_{s}\left(N^{2}K+N^{3}\right))$; here $N_{s}$ is the number of testing samples. The O(·) is the Big O notation for asymptotic computational complexity analysis [49].

## 4. Results and Discussion

In this section numerical results are presented. First, the performance of deep learning-based trained ADMM adaptive thresholding is evaluated with a generic real-valued Gaussian model, and next, a complex-valued SFCW radar model given in Section 2.1 is used.

### 4.1. Generic Gaussian Model

In this subsection, our proposed approach is evaluated using the generic Gaussian data. The order of this subsection is summarized as follows. First, the performance of the proposed approach is compared with state-of-the-art approaches for 50% and 25% compression ratios. Second, the Cramér–Rao bound (CRB) of unbiased estimation of low-rank and sparse matrices is used to evaluate the proposed approach. Third, to investigate the robustness of the proposed approach, two scenarios are used: (a) testing SNR uncertainty and (b) deviation in measurement matrices $A_{l}$ and $A_{s}$ between training and testing. Fourth, the performance comparison between ADMM- and FISTA-based approaches for RPCA is evaluated. Here, the approach given in [30] (CORONA) is used as unfolded FISTA-based approach for RPCA.

In the generic Gaussian model, the elements of $A_{l}\;=\;A_{s}\;=\;A\in R^{K\times MN}$ are generated once from an i.i.d. Gaussian with zero mean and unit variance. In this work, training and testing data are synthetically generated based on the system model given in (1). Therefore, ground-truth low-rank and sparse matrices are available in the training phase. In case only the received data vector y in (1) is available, in general, Algorithm 1 or LRPSRC given in (8) can be used to generate low-rank and sparse matrices in the training phase. Let the received signal, noise vector, and low-rank and sparse matrices for the *i*-th data sample as given in (1) be denoted by $y_{i}\in R^{K}$, $n_{i}\in R^{K}$, $L_{i}\in R^{M\times N}$, and $S_{i}\in R^{M\times N}$, respectively. We generate a low-rank matrix $L_{i}$ with rank *r* as $L_{i}=G_{i}H_{i}^{T}$ with $G_{i}\in R^{M\times r}$ and $H_{i}\in R^{N\times r}$. Here, elements of $G_{i}$, $H_{i}$ and non-zero entries of $S_{i}$ are generated independently from an i.i.d. Gaussian with zero mean and unit variance. The fixed number of non-zero locations of each $S_{i}$ are selected uniformly. We normalized $S_{i}$ and $L_{i}$ to have a unit Frobenius norm (i.e., ${∥L_{i}∥}_{F}^{2}={∥S_{i}∥}_{F}^{2}=1$). For better readability, we introduce a parameter set as $P=\{M,N,T_{s},N_{s},L_{p},L_{w},E_{p},r,{∥S_{i}∥}_{0},{\mathrm{SNR}}_{\mathrm{tr}},{\mathrm{SNR}}_{t}\}$. Here, $T_{s}$, $N_{s}$, $E_{p}$, *r*, ${∥S_{i}∥}_{0}$, ${\mathrm{SNR}}_{\mathrm{tr}}$, and ${\mathrm{SNR}}_{t}$ are the number of training samples, number of testing samples, number of epochs, rank of the low-rank matrix, number of non-zero elements of the sparse matrix, SNR of the training data, and SNR of the testing data, respectively. The signal-to-noise ratio (SNR) of the *i*-th data sample for given A is defined as $\mathrm{SNR}:={∥A\mathrm{vec}(L_{i}+S_{i})∥}_{2}^{2}/{∥n_{i}∥}_{2}^{2}$. First, we generate a Gaussian noise vector $n_{i}$, then re-normalize $n_{i}$ to reach a given target SNR, and we set the same SNR for all samples.

In the training stage, we set different learning rates denoted by $L_{w}$ and $L_{p}$ for the weights of the linear layers ($W_{1},W_{2},W_{3},W_{4}$) and other parameters ($\lambda_{S}^{t}$, $\lambda_{L}^{t},\gamma^{t},\rho^{t}$) given in Θ. The main objective for setting different learning rates is to reduce over-fitting to training data. Generally, many training samples are required to train a deep neural network. However, due to the specific architecture of the iterative algorithm, we are able to train the DNN with a small data set with the number of training samples $T_{s}=500$. In the training phase, the adaptive moment estimation (Adam) optimizer [50] is used to train the DNN. Here, we initialize $W_{1}^{t}=A_{l}^{†}$, $W_{2}^{t}=A_{s}$, $W_{3}^{t}=A_{s}^{†}$, and $W_{4}^{t}=A_{l}$ to mimic the ADMM Algorithm 1 and γ=1. In the inference phase, to evaluate the performance of the DNN, the normalized average root mean squared error is used. For the low-rank and sparse matrix, it is given by

(23) $$\begin{array}{cc} {\mathrm{NRMSE}}_{L} & =\frac{1}{N_{s}}\sum_{i=1}^{N_{s}}\left(\frac{{∥{\hat{L}}_{i}-L_{i}∥}_{F}}{{∥L_{i}∥}_{F}}\right), \end{array}$$

(24) $$\begin{array}{cc} {\mathrm{NRMSE}}_{S} & =\frac{1}{N_{s}}\sum_{i=1}^{N_{s}}\left(\frac{{∥{\hat{S}}_{i}-S_{i}∥}_{F}}{{∥S_{i}∥}_{F}}\right). \end{array}$$

The outputs of a DNN with *T* layers for the *i*-th testing sample are given by ${\hat{S}}_{i}$ and ${\hat{L}}_{i}$, respectively. The CRB given in (A4) is based on the combined recovery error of both low-rank and sparse matrices. The combined average mean squared error and the combined average normalized root mean squared error for both low-rank and sparse matrices are given by

(25) $$\begin{array}{cc} {\mathrm{MSE}}_{\mathrm{LS}} & =\frac{1}{N_{s}}\sum_{i=1}^{N_{s}}\left({∥x_{i}-{\hat{x}}_{i}∥}_{2}^{2}\right), \end{array}$$

(26) $$\begin{array}{cc} {\mathrm{NRMSE}}_{\mathrm{LS}} & =\frac{1}{N_{s}}\sum_{i=1}^{N_{s}}\left(\frac{{∥x_{i}-{\hat{x}}_{i}∥}_{2}}{{∥x_{i}∥}_{2}}\right), \end{array}$$

in which $x_{i}={\left[\mathrm{vec}{\left(L_{i}\right)}^{T}\;\mathrm{vec}{\left(S_{i}\right)}^{T}\right]}^{T}$ and ${\hat{x}}_{i}={\left[\mathrm{vec}{\left({\hat{L}}_{i}\right)}^{T}\;\mathrm{vec}{\left({\hat{S}}_{i}\right)}^{T}\right]}^{T}$.

Both Algorithm 1 and LRPSRC given in (8) are implemented using Matlab [51], and LRPSRC is solved using the CVX package [52]. Notice that, in the LRPSRC, $\lambda_{l}$ and $\lambda_{s}$ are set to 1 and $1/\sqrt{\max(M,N)}$, respectively, as suggested by [17]. Note that for Algorithm 1, there is no specific rule to select the $\lambda_{l}$ and $\lambda_{s}$ and ρ, thus they are manually tuned based on data. When A is identity matrix, there is a specific rule to select $\lambda_{s}$ as $1/\sqrt{\max(M,N)}$ [17]. Note that, as a rule of thumb, thresholding parameters $\lambda_{S}$ and $\lambda_{L}$ given in (17) and (18) are initialized as $\lambda_{S}=\lambda_{s}/\rho$ and $\lambda_{L}=\lambda_{l}/\rho$, respectively [17]. The ADMM penalty factor ρ has an important impact on the convergence of the Algorithm 1. Usually, as ρ increases, the algorithm converges faster. However, ρ cannot be arbitrarily large, as it may overshoot the algorithm. Furthermore, ρ should not be too big or too small. However, finding an optimal value for ρ is an open problem, and it depends on the application/data. As a rule of thumb, ρ can be set as $0.25MN/{∥y∥}_{1}$ [27]. In this work, we set $\rho =10/\sqrt{\max(M,N)}$, as we observed that the value suggested in [27] is not optimal for our data. Note that, unless otherwise stated, for all the simulation with Gaussian data, aforementioned parameter settings are used throughout this paper. The Pytorch package was used to implement the DNN [53].

**Figure 3:**
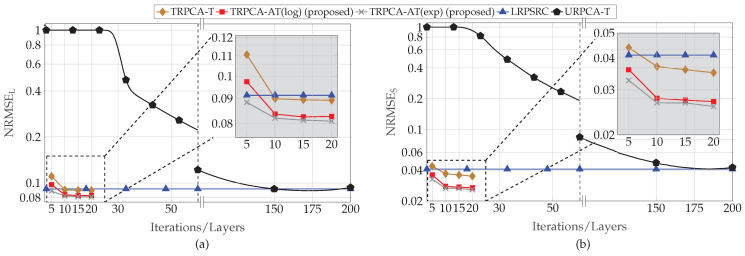
Average recovery error of low-rank (**a**) and sparsity (**b**) contributions for compression ratio K/MN=50% for ADMM-based trained RPCA with thresholding (TRPCA-T), proposed ADMM-based trained RPCA with adaptive thresholding based on logarithm heuristic (TRPCA-AT(log)), proposed ADMM-based trained RPCA with adaptive thresholding based on exponential heuristic (TRPCA-AT(exp)), low-rank plus sparse recovery with convex relaxation (LRPSRC), and ADMM-based untrained RPCA with thresholding (URPCA-T).

**Figure 4:**
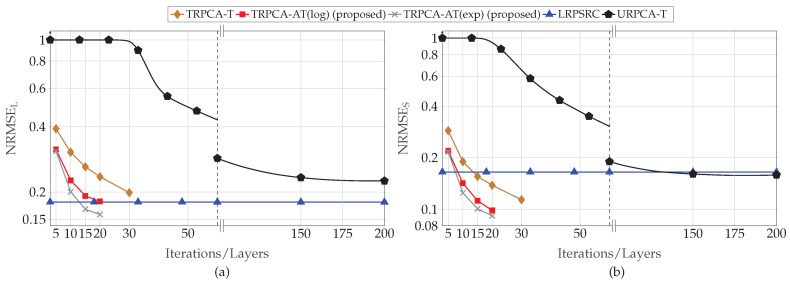
Average recovery error of low-rank (**a**) and sparsity (**b**) contributions for compression ratio K/MN=25% for ADMM-based trained RPCA with thresholding (TRPCA-T), proposed ADMM-based trained RPCA with adaptive thresholding based on logarithm heuristic (TRPCA-AT(log)), proposed ADMM-based trained RPCA with adaptive thresholding based on exponential heuristic (TRPCA-AT(exp)), low-rank plus sparse recovery with convex relaxation (LRPSRC), and ADMM-based untrained RPCA with thresholding (URPCA-T).

First, we analyzed the performance of the proposed approach for different compression ratios (K/MN) with respect to the number of layers of the DNN. For this simulation, the parameter set *P* is given by P={30,30,500,1200,0.1,0,500,2,5,20dB,20dB}. Here, DNN only learns $\lambda_{S}^{t}$, $\lambda_{L}^{t}$, and $\rho^{t}$ instead of all the parameters given in Θ, i.e., $L_{w}=0$. This is due to the fact that the performance gain improvement by learning all the parameters given in Θ is very small compared to learning only $\lambda_{S}^{t}$ and $\lambda_{L}^{t}$. The average normalized RMSEs for the different number of layers of the DNN are, for compression ratio (K/MN) 50% and 25%, shown in Figure 3 and Figure 4, respectively. Figure 3 and Figure 4 show that the proposed DNN-based thresholding (TRPCA-AT(log) and TRPCA-AT(exp)) outperforms the URPCA-T and the LRPSRC. Further, it is observed that as the number of layers increases, the average NRMSE decreases. For 50% compression ratio, the average NRMSE does not show a large variance after ten layers. However, for 25%, this is not the case. This is due to the fact that, as the compression increases, recovering of the low-rank and the sparse matrices becomes more challenging.

Further, the TRPCA-AT outperforms the TRPCA-T. This performance improvement is mainly due to the iterative reweighting of $\ell_{1}$-norm and nuclear norm minimization. In addition, the improvement over unweighted to iterative reweighting is more visible as the compression increases (i.e., as the problem gets more challenging). As an example, for 25% compression ratio, the average NRMSE improvement between the TRPCA-T with twenty layers and TRPCA-AT(exp) with twenty layers for the low-rank and sparse components are 32.93% and 50.77%, respectively. However, for 50% compression ratio, this improvement for the low-rank and sparse components are 9.31% and 26.21%, respectively. Further, we observe slight performance gains as the decay function is changed from log-determinant to exponential.

Next, we analyzed the convergence speed of the proposed TRPCA-AT(exp) and TRPCA-AT(log) with URPCA-T. For 50% of compression ratio, TRPCA-AT with ten layers outperforms URPCA-T with 150 iterations. Therefore, in the testing phase (inference phase), our proposed approaches (TRPCA-AT(exp) and TRPCA-AT(log)) are fifteen times faster than the conventional untrained approach URPCA-T. Moreover, for 25% of compression ratio, TRPCA-AT with twenty layers outperforms URPCA-T with 150 iterations. Thus, our approach is 7.5 times faster than the untrained approach URPCA-T. It is worth noticing that one layer of the DNN of our proposed approach is equivalent to one iteration of the conventional untrained approach URPCA-T. Therefore, the proposed approaches (TRPCA-AT(exp) and TRPCA-AT(log)) achieve lower NRMSE than the untrained approach URPCA-T with a much lower number of iterations. In Table 1, NRMSEs of recovered low-rank and sparse matrices with the corresponding number of iterations are listed for comparison.

**Figure 5:**
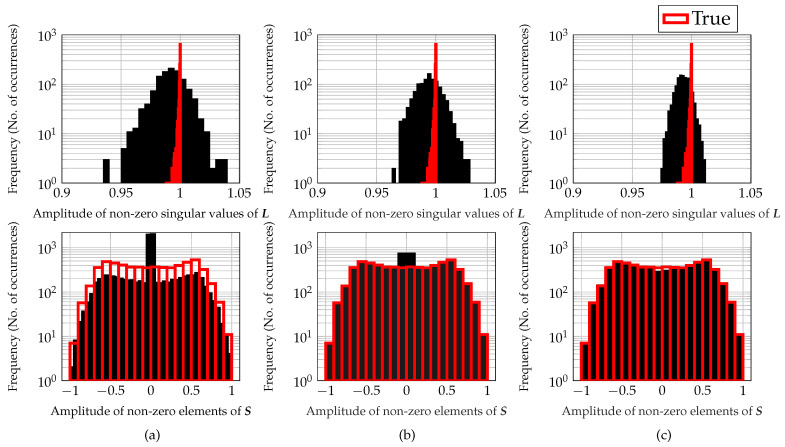
Histograms of the non-zero singular values of L (**top**) and non-zero elements of S (**bottom**) for K/MN=50%. (**a**) TRPCA-T, (**b**) TRPCA-AT(log) (proposed) and (**c**) TRPCA-AT(exp) (proposed). Note that, In the figure, true histograms are shown in red color and the recovered histograms are shown in black color. It is noticeable that the proposed non-convex iterative reweighted approaches (TRPCA-AT(exp) and TRPCA-AT(log)) closely follow the histograms of the true non-zero elements of S and non-zero singular values of L compared to the unweighted approach TRPCA-T. In addition, the recovered S by the unweighted approach TRPCA-T has many small values compared to the iterative reweighted approaches, i.e., the iterative reweighted approach achieves a more sparse solution than the unweighted approach.

To further demonstrate the advantage of non-convex iterative reweighting of $\ell_{1}$-norm and nuclear norm minimization, histograms of the non-zero singular values of the low-rank matrix and non-zero element of the sparse matrix are shown in Figure 5 for the DNN with 20 layers. Here, these histograms correspond to the simulation given in Figure 3, i.e., 1200 testing samples and compression ratio K/MN=50%. Based on Figure 5, for the sparse matrix S, the proposed non-convex iterative reweighted approaches (TRPCA-AT(exp) and TRPCA-AT(log)) closely follow the histogram of the true sparse matrix. Moreover, for a given value range, the number of occurrences of the recovered sparse matrix by the unweighted approach TRPCA-T is less than the true number of occurrences of that value range as shown in Figure 5a. However, this is not the case for the non-convex iterative reweighted approaches (TRPCA-AT(exp) and TRPCA-AT(log)). These results validate that the important features preserved by the large coefficients are well recovered by the iterative reweighted approaches. This is the reason for the performance improvement of the iterative reweighted approaches compared to the unweighted approach TRPCA-T. In addition, recovered sparse matrices by the unweighted approach TRPCA-T have many small values compared to the iterative reweighted approaches. This indicates that the iterative reweighted approaches achieve more sparse solution than the unweighted approach.

As seen in Figure 5, histograms of the non-zero singular values of the low-rank matrix by the proposed non-convex iterative reweighted approaches are less spread out compared to the histogram of the unweighted approach TRPCA-T. This also validates the aforementioned argument that important features preserved by the large coefficients are well recovered by the iterative reweighted approaches TRPCA-AT(exp) and TRPCA-AT(log). Note that in the histograms, the number of occurrences of zero value is not shown. This is due to the fact that the number of occurrences of zero value is much larger than occurrences of other values. In Figure 5, histograms corresponding to the compression ratio K/MN=50% are shown, and for the compression ratio K/MN=25%, similar results were observed.

#### 4.1.1. Cramér–Rao Bound (CRB) Analysis

To further evaluate the performance of the proposed approach, the Cramér-Rao bound (CRB) of unbiased estimation of low-rank and sparse matrices given in [18] (Equation (A4)) is used. For completeness, the CRB and recovery guarantees of the RPCA are given in Appendix A.3. Note that in [18], the measurement matrices $A_{l}$ and $A_{s}$ are assumed to be a selection operator. Therefore, to have a fair comparison, first we consider that both $A_{l}$ and $A_{s}$ are identity matrices, i.e., standard RPCA problems. Now, the data acquisition model (Equation (1)) is simplified as

(27) Y=L+S+N,

where Y and N are received signal matrix and noise matrix of size M×N, respectively. For this simulation, parameter set *P* is given by $\left\{30,30,500,1200,1\times 10^{-2},/5\times 10^{-4},10,2,5,\left[-5:5:20\right]\;\mathrm{dB},\left[-5:5:20\right]\;\mathrm{dB}\right\}$. In this simulation, we set the number of layers of the DNN as 10.

**Figure 6:**
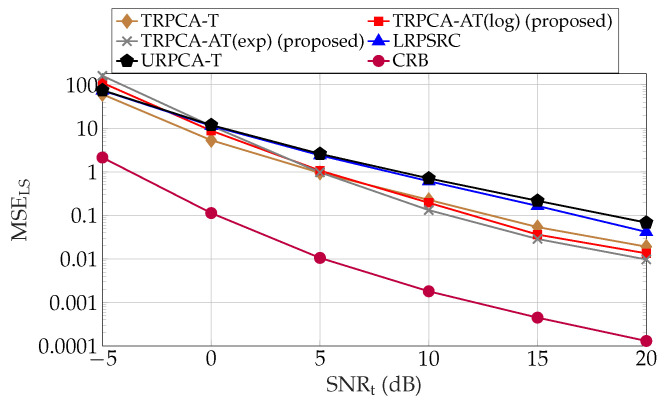
Average combined recovery error of low-rank and sparse matrices as given in (25) and Cramér-Rao bounds as given in (A4) for compression ratio K/MN=100% for ADMM-based trained RPCA with thresholding (TRPCA-T), proposed ADMM-based trained RPCA with adaptive thresholding based on logarithm heuristic (TRPCA-AT(log)), proposed ADMM-based trained RPCA with adaptive thresholding based on exponential heuristic (TRPCA-AT(exp)), low-rank plus sparse recovery with convex relaxation (LRPSRC), and ADMM-based untrained RPCA with thresholding (URPCA-T).

Figure 6 shows the CRB and average MSE of the combined low-rank and sparse matrices for 1200 testing samples for different SNR levels ranging from −5 dB to 20 dB in steps of 5 dB. Here, we consider same SNR in both training and testing. As an example, if the testing SNR (${\mathrm{SNR}}_{t}$) is 20 dB, then training SNR (${\mathrm{SNR}}_{\mathrm{tr}}$) is 20 dB. As per Figure 6, it can be seen that the non-convex approach TRPCA-AT has the best performance compared to other approaches in higher SNR regime. Note that, here, the performance gap between the non-convex approach TRPCA-AT and the non-convex approach TRPCA-T is small. This is due to the fact that, as observed in Figure 3 and Figure 4, when compression decreases, the gain achieve by the non-convex approaches decreases.

**Figure 7:**
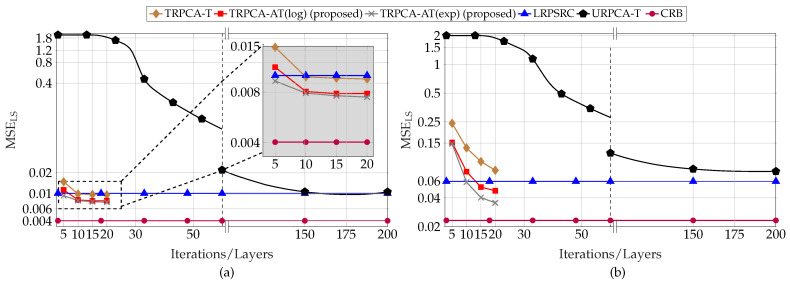
Average combined recovery error of low-rank and sparse matrices as given in (25) and Cramér-Rao bounds as given in (A4) for compression ratio K/MN=50% (**a**) and K/MN=25% (**b**) for ADMM-based trained RPCA with thresholding (TRPCA-T), proposed ADMM-based trained RPCA with adaptive thresholding based on logarithm heuristic (TRPCA-AT(log)), proposed ADMM-based trained RPCA with adaptive thresholding based on exponential heuristic (TRPCA-AT(exp)), low-rank plus sparse recovery with convex relaxation (LRPSRC), and ADMM-based untrained RPCA with thresholding (URPCA-T).

Next, we compare the results shown in Figure 3 and Figure 4 with the CRB given in [18] (Equation (A4)). In [18], the measurement matrix A is assumed to be a selection operator which selects a random subset of size *K* from MN entries. Since this is the closest matching CRB to our model given in (1), we have considered this formulation as a benchmark. Further, we consider that A is fixed over all testing samples. Figure 7 shows the CRB of the combined low-rank and sparse matrices for compression ratios 50% and 25%. It can be seen that the non-convex approach TRPCA-AT has the closest performance to the CRB. As the compression increases, the estimation of low-rank and sparse matrices from compressive measurements becomes more challenging. This can be seen by the increase of CRB as the compression ratio changes from 50% to 25%.

#### 4.1.2. Robustness of the Proposed Approach

We considered two scenarios to analyze the robustness of the proposed trained ADMM adaptive thresholding approaches TRPCA-AT and TRPCA-T. First, motivated by [54], we analyzed the performance with respect to the test SNR uncertainty, i.e., the SNRs of training phase and testing phase are different. Second, we analyzed the effect of deviations in the measurement matrices $A_{l}$ and $A_{s}$ in (1) between training and testing.

**Figure 8:**
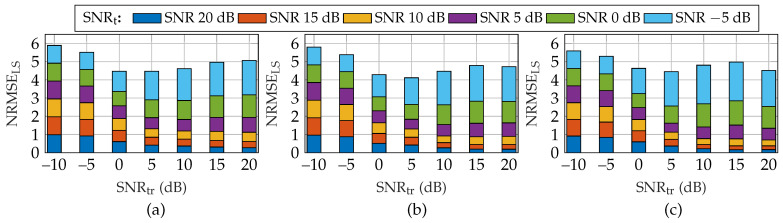
Average combined recovery error of low-rank and sparse matrices for compression ratio K/MN=50% for training at a single SNR and testing with different SNRs for (**a**) ADMM-based trained RPCA with thresholding (TRPCA-T), (**b**) the proposed ADMM-based trained RPCA with adaptive thresholding based on logarithm heuristic (TRPCA-AT(log)), and (**c**) the proposed ADMM-based trained RPCA with adaptive thresholding based on exponential heuristic (TRPCA-AT(exp)). In the presence of uncertainty about testing SNR, then training with an SNR ≈5 dB is favorable.

To this end, to evaluate the effect of testing SNR uncertainty, training SNR (${\mathrm{SNR}}_{\mathrm{tr}}$) is changed from −10 dB to 20 dB with a step size of 5 dB. In addition, testing SNR (${\mathrm{SNR}}_{t}$) is changed from −5 dB to 20 dB with a step size of 5 dB. For this simulation, *P* is given by $\left\{30,30,500,1200,1\times 10^{-1},5\times 10^{-4},20,2,5,\left[-10:5:20\right]\;\mathrm{dB},\left[-5:5:20\right]\;\mathrm{dB}\right\}$. The cumulative average ${\mathrm{NRMSE}}_{\mathrm{LS}}$ (where ${\mathrm{NRMSE}}_{\mathrm{LS}}$ is defined in (26)) over all testing SNRs for each training SNR is shown in Figure 8. Here, we set K/MN=50% and number of layers of the DNN as 10. We trained the DNN for 20 epochs using the Adam. Based on the results shown in Figure 8, we observed that, for all three approaches (TRPCA-AT(log), TRPCA-AT(exp), TRPCA-T), the cumulative average ${\mathrm{NRMSE}}_{\mathrm{LS}}$ decreases as training SNR increases to some extent, and then, again, the cumulative average increases as training SNR further increases. Hence, these results show the importance of knowing the testing SNR, and as a simple rule, training SNR should be same as testing SNR to achieve the best performance. On the other hand, training with an SNR ≈5 dB is favorable in the presence of uncertainty about testing SNR.

**Figure 9:**
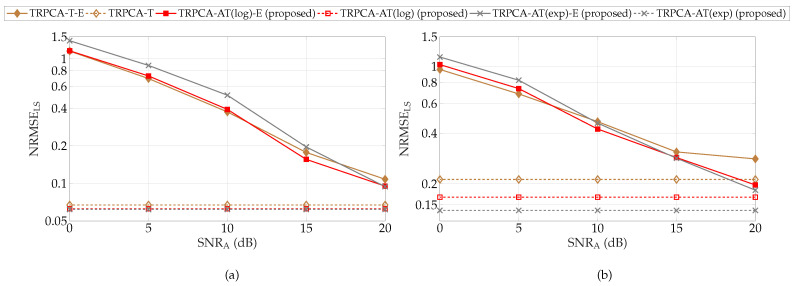
Average combined recovery error of low-rank and sparse matrices for compression ratio K/MN=50% (**a**) and K/MN=25% (**b**) with different model error levels for ADMM-based trained RPCA with thresholding (TRPCA-T), proposed ADMM-based trained RPCA with adaptive thresholding based on logarithm heuristic (TRPCA-AT(log)), and proposed ADMM-based trained RPCA with adaptive thresholding based on exponential heuristic (TRPCA-AT(exp)). Here, model error means that the train and testing samples are generated with different measurement matrices. For training, y=Avec(L+S)+n; for testing, $y=\bar{A}\mathrm{vec}\left(L+S\right)+n$ with $\bar{A}=A+E$. The results with model error are represented by solid lines, whereas dotted lines indicates the results without model error ($E=0_{K,MN}$).

**Figure 10:**
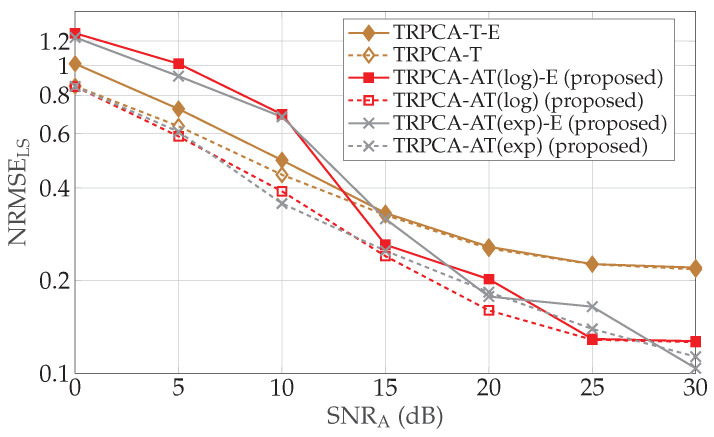
Average combined recovery error of low-rank and sparse matrices for compression ratio K/MN=25% with different model error levels for ADMM-based trained RPCA with thresholding (TRPCA-T), proposed ADMM-based trained RPCA with adaptive thresholding based on logarithm heuristic (TRPCA-AT(log)), and proposed ADMM-based trained RPCA with adaptive thresholding based on exponential heuristic (TRPCA-AT(exp)). Here, model error means that the training and testing samples are generated with different measurement matrices. For training, y=Avec(L+S)+n; for testing, $y=\bar{A}\mathrm{vec}\left(L+S\right)+n$ with $\bar{A}=A+E$. The results with model error are represented by solid lines, whereas dotted lines indicates the results when model error distribution is included in training.

Next, we evaluate the performance of the proposed approaches for different measurement matrices $A_{l}$ and $A_{s}$ in (1) during training and testing. For simplicity, we assume that $A_{l}\;=\;A_{s}\;=\;A\;\in \;R^{K\times MN}$. In the training phase, y=Avec(L+S)+n while in the testing phase $y=\bar{A}\mathrm{vec}\left(L+S\right)+n$. Here, $\bar{A}=A+E\in \;R^{K\times MN}$ is the measurement matrix with error and $E\;\in R^{K\times MN}$. To quantify the effect of E, ${\mathrm{SNR}}_{A}:={∥\mathrm{vec}(A)∥}_{2}^{2}/{∥\mathrm{vec}(E)∥}_{2}^{2}$ is used as a metric. We evaluate the performance of the proposed approaches while changing ${\mathrm{SNR}}_{A}$ from 0 dB to 20 dB in steps of 5 dB. For this simulation, parameter set *P* is given by $\left\{30,30,500,1200,1\times 10^{-1},1\times 10^{-6},50,2,5,20\;\mathrm{dB},{\mathrm{SNR}}_{t}\right\}$. Note that testing SNR varies as ${\mathrm{SNR}}_{A}$ changes, therefore, it is shown as ${\mathrm{SNR}}_{t}$ in parameter set *P*. Figure 9 shows the average NRMSEs of the combined low-rank and sparse matrices K/MN=50% and 25%. In Figure 9, solid lines represent the NRMSEs for the model with measurement matrix error ($\bar{A}=A+E$); we also include a prefix “-E” in the legend of the figure to indicate it. In Figure 9, the dotted line shows the NRMSEs without error in the measurement matrix. Based on the results shown in Figure 9, the proposed approaches are robust for smaller deviations like ${\mathrm{SNR}}_{A}=20$ and 15 dB. However, for higher deviations ${\mathrm{SNR}}_{A}$≤10 dB, the proposed approaches are not robust enough and additional measures are required to rectify the matrix deviation. As a countermeasure, we assume that the model error distribution is available in training as well. Here, both *i*-th sample of training and testing data are generated by $y_{i}=\bar{A}\mathrm{vec}\left(L_{i}+S_{i}\right)+n_{i}$. For training, $\bar{A}=A+E_{tr,i}$, and for testing, $\bar{A}=A+E_{t,i}$. Here, for each training and testing sample, $E_{tr,i}$ and $E_{t,i}$ are generated independently from an i.i.d. Gaussian with zero mean and unit variance. For comparison, we include results with training without error distribution, i.e., training data are generated as $y_{i}=A\mathrm{vec}\left(L_{i}+S_{i}\right)+n_{i}$ while keeping testing data the same, i.e., $y_{i}=\bar{A}\mathrm{vec}\left(L_{i}+S_{i}\right)+n_{i}$. Note that this result is shown as solid lines in Figure 10. As shown in Figure 10, when model error distribution is included in training (dotted line in Figure 10), the NRMSEs show improvement over training without distribution of E when ${\mathrm{SNR}}_{A}$ is in the range of 0 dB to 15 dB. Moreover, as ${\mathrm{SNR}}_{A}$ increases, i.e., deviation decreases, training without distribution of error E provides similar results as training with distribution of E. As a conclusion, when there is high deviation in the measurement matrix, a robust training approach, i.e., training with distribution of E, provides an advantage.

#### 4.1.3. ADMM or FISTA to Solve RPCA Problem

**Figure 11:**
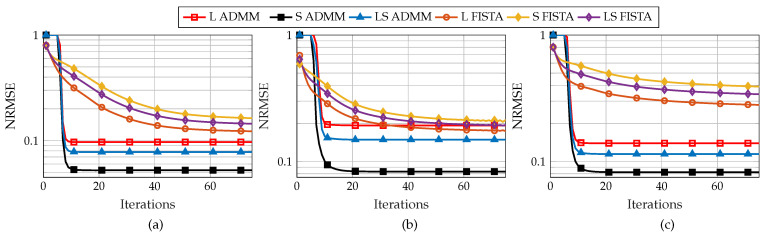
Average NRMSE of low-rank and sparsity contributions for K/MN=100% for ADMM- and FISTA-based approaches. (**a**) rank(L)=1 and $p_{s}=0.1$, (**b**) rank(L)=2 and $p_{s}=0.1$ and (**c**) rank(L)=2 and $p_{s}=0.2$. The ADMM-based approach achieves a lower NRMSE with a lower number of iterations compared to the FISTA-based approach.

In this work, we consider an iterative algorithm based on the ADMM [39] to solve the RPCA problem. Alternatively, other methods such as ISTA and FISTA can be used [30,37]. In the following, we first compare the performance of the untrained ADMM-based Algorithm 1 with $g_{s}\left(x\right)\;=\;g_{l}\left(x\right)\;=\;1$ (URPCA-T) and the untrained algorithm based on FISTA as given in [30,37]. Further, we consider three different combinations for the rank of L and sparsity of S with ${∥S∥}_{0}=MNp_{s}$. The aforementioned three combinations are given by rank(L)={1,2,2} and $p_{s}=\left\{0.1,0.1,0.2\right\}$. Here, we consider 250 test samples in each combination. It turns out that for all three combinations, the ADMM-based approach achieves lower NRMSEs with fewer numbers of iterations compared to the FISTA, as shown in Figure 11. In this simulation, we consider standard RPCA where $A_{l}$, $A_{s}$ in (1) are equal to the identity matrix. We chose this scenario because it is the simplest non-compression scenario. Note that, for FISTA, soft-thresholding and singular value thresholding parameters are set as 0.05 and 0.1max(σ(Y)), in which σ(Y) are the singular values of Y.

#### 4.1.4. Performance Evaluation for Experimental Ultrasound Imaging Data

To further assess the performance of ADMM- and FISTA-based approaches in the context of algorithm unfolding, we consider the FISTA-based unfolded approach in [30] (CORONA). For fair comparison, we consider two types of data: (a) experimental ultrasound imaging data used in [30] (available at https://www.wisdom.weizmann.ac.il/yonina accessed on 20 December 2021) and (b) complex-valued generic Gaussian data. Note that for the generic data, we set M=1024 and N=20 to match the same dimension as ultrasound data in [30]. Moreover, real and complex valued entries of both low-rank and sparse matrices are generated independently from an i.i.d. Gaussian with zero mean and unit variance. Further, the fixed number of non-zero locations of each $S_{i}$ are selected uniformly. The rank of each $L_{i}$ is set as 2, and the number of non-zero elements of each $S_{i}$ is set as 0.01MN. We normalized $S_{i}$ and $L_{i}$ to have a unit Frobenius norm (i.e., ${∥L_{i}∥}_{F}^{2}={∥S_{i}∥}_{F}^{2}=1$). Further, SNR during training and testing is 20 dB. To have a fair comparison, we use the same number of layers as in CORONA. Both CORONA and our approaches are trained using the Adam with 20 epochs. For CORONA, the same settings as in [30] were used. We utilized the CORONA implementation from the author’s website (https://www.wisdom.weizmann.ac.il/yonina accessed on 20 December 2021). Note that experimental ultrasound data in [30] follows the standard RPCA problem: $A_{l}$, $A_{s}$ in (1) are equal to identity matrix. Therefore, for comparison, our ADMM-based approach is implemented without linear layers, i.e, all $W_{1}^{t}$, $W_{2}^{t}$, $W_{3}^{t}$, and $W_{4}^{t}$ are identity matrices, i.e., aforementioned linear layers are omitted from the DNN. Thus, our proposed approach only learns the thresholding parameters $\lambda_{S}^{t},\;\lambda_{L}^{t},\;\gamma^{t}$, and $\rho^{t}$ with a learning rate of $L_{p}=5\times 10^{-4}$. Note that in this setting, our proposed approach is only required to learn the four parameters per layer, i.e., 40 parameters for a DNN with ten layers. However, in CORONA, for a single layer, six convolutional weights matrices and two thresholding parameters have to be learned. Based on convolutional filter sizes given in [30], CORONA with ten layers is required to learn 6×{3×(5×5)+7×(3×3)}+20=848 parameters compared to 40 parameters in our approach.

First, we compared our proposed approach with CORONA for experimental ultrasound data, and corresponding results are shown in Table 2. The experimental ultrasound data in [30] consists of 2400 training samples and 800 testing samples. For performance comparison, similar to [30], average MSE is utilized as a metric for ultrasound data. Note that for experimental ultrasound data, CORONA shows slightly better performance in recovering S, compared to the proposed approaches. For the low-rank matrix L recovery, our proposed approaches and CORONA show similar performance levels. For the sparse matrix S recovery, our proposed approaches show slightly worse performances compared to the CORONA. This is to be expected since, in CORONA, $\ell_{1,2}$-norm minimization is used for S, which reflects the row-sparse nature of the experimental ultrasound data. Our approach is formulated for plain unstructured sparsity in the matrix, and it is, therefore, not that optimized for sparsity patterns in experimental ultrasound data. Note that it is also straightforward to modify our ADMM approaches for soft-thresholding related to the $\ell_{1,2}$-norm.

Next, we compared CORONA and our approach for the generic Gaussian data. For the Gaussian data set, we consider 2400 training samples and 1600 testing samples. Here, our proposed approach outperforms the CORONA, as shown in Table 3. This is due to the fact that the data acquisition model given in (27) follows an unstructured sparsity model and does not include convolution operation. Thus, since the generic Gaussian data does not follow the sparsity model as the ultrasound data in [30], the performance of CORONA is degraded compared to our approach. As discussed above, the ultrasound data follows the standard RPCA where there is no compression, i.e., $A_{l}$, $A_{s}$ in (1) are equal to identity matrix. In order to evaluate our approach on compressed data, we manually applied the compression on ultrasound data as discussed next.

**Figure 12:**
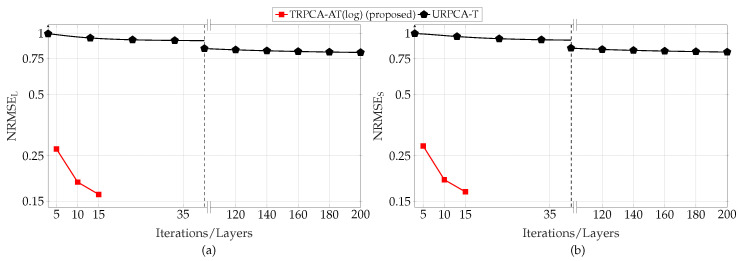
Average NRMSE of low-rank (**a**) and sparsity (**b**) contributions for experimental ultrasounds data with 50% compression ratio for the proposed ADMM-based trained RPCA with adaptive thresholding based on logarithm heuristic (TRPCA-AT(log)) and ADMM-based untrained RPCA with thresholding (URPCA-T).

The received signal matrix of ultrasound data $Y\;\in C^{M\times N}$ is given by Y=L+S+N, where L, S, and N are low-rank, sparse, and noise matrices of size M×N, respectively. In ultrasound data, a single measurement consists of twenty frames of size 32×32; this results in M=20 and N=1024. Lets denote the frame size as m×n. In order to evaluate our approach to compressed data, we manually applied the compression on ultrasound data by using a Gaussian matrix A which compresses a 32×32 frame to a 16×16 frame, i.e., 50% compression. In more detail, the matrix A is a linear operator which maps the vector space $C^{mn}$ to vector space $C^{k}$. We set mn=1024 and k=512. Now, after the compression, the received signal for a single measurement is given by $Y_{cs}\;\in C^{M\times k}$, i.e., 20×512. Here, we consider 1800 training samples and 400 testing samples. We train our proposed approach using the Adam optimizer with 20 epochs with learning rate of $1\times 10^{-4}$. The average normalized RMSEs for the different numbers of layers of the DNN for k/mn=50% is shown in Figure 12. As shown in Figure 12, our proposed approach TRPCA-AT(log) outperforms the untrained approach URPCA-T in terms of NRMSE as well as the number of iterations. The proposed approach TRPCA-AT(log) is able to achieve much lower NRMSE by only using 15 layers compared to the 200 iterations in the untrained approach URPCA-T. Therefore, our approach is 13 times faster than the untrained approach.

### 4.2. SFCW Radar Model

In this subsection, the performance evaluation of the ADMM-based trained RPCA with adaptive thresholding is now performed for the SFCW radar model given in Section 2.1. In the simulations, we set the carrier frequency $f_{c}$ of 300 GHz and bandwidth *B* as 5 GHz. Here, we consider two types of simulations: (a) small scale and (b) large scale. For the small scale, we consider N=M=30, i.e., 30 antennas and 30 frequency bands. Both height and length of the layered structure are 0.5 m. In the simulations, we consider six defects, and the scene is partitioned into a 16×16 grid with equal grid size (i.e., Q=256). The grid size is selected according to the Rayleigh resolution of the radar. For the large scale, we consider N=M=100, i.e., 100 antennas and 100 frequency bands. In addition to that, we have increase both height and length of the layered structure to 2.5 m. This results in an increase of the grid size, and the grid size for this scenario is 83×83, (i.e., Q=6889). Moreover, here we consider nine defects in the radar scene.. The inter-antenna spacing is chosen as half of the wavelength of $f_{c}$. We consider a single-layered structure, and the distance to the front surface of the layered structure is 1.0 m. Denote the reflection of the layered material structure, noise matrix, and sparse vector for the *i*-th data sample, given in (7), by $Y_{i}^{l}$, $Z_{i}$ and $s_{i}\;\forall \;i$, respectively. In the simulations, the signal-to-noise ratio of the *i*-th data sample for given Φ and D is defined as $\mathrm{SNR}\;:=\;{∥\Phi \mathrm{vec}\left(Y_{i}^{l}\right)\;+\;\Phi Ds_{i}∥}_{2}^{2}/{∥\Phi \mathrm{vec}(Z_{i})∥}_{2}^{2}\;=\;20$ dB. Here, we set same SNR for all samples. Note that the SFCW data consists of complex numbers, thus, in this work, we implemented the DNN which supports complex numbers by using the PyTorch version 1.8.1 [53]. Here, we initialize $W_{1}^{t}=A_{l}^{H}$, $W_{2}^{t}=A_{s}$, $W_{3}^{t}=A_{s}^{H}$, and $W_{4}^{t}=A_{l}$ to mimic the ADMM Algorithm 1.

Interestingly, in contrast to the generic Gaussian model, only learning the $\lambda_{S}^{t}$ and $\lambda_{L}^{t}$ does not achieve satisfactory average NRMSEs of the low-rank and sparse components. Therefore, we enable learning all parameters given in Θ. Further, we notice that the stochastic gradient descent (SGD) [55] performs better than the Adam in learning all the parameters given in Θ together. Therefore, we consider a three-stage training process for better learning. A detailed breakdown of this three-stage training process is given in Appendix A.4.

For defect detection by SFCW radar, we considered a data set of 600 samples. Here, 500 data samples are used for training and validation, and 100 data samples are used for testing. We used Matlab [51] to generate the SFCW data based on (7). First, we present the results related to the small-scale simulations. Next, results related to the large-scale simulations are presented.

#### 4.2.1. SFCW Small-Scale Simulations

**Figure 13:**
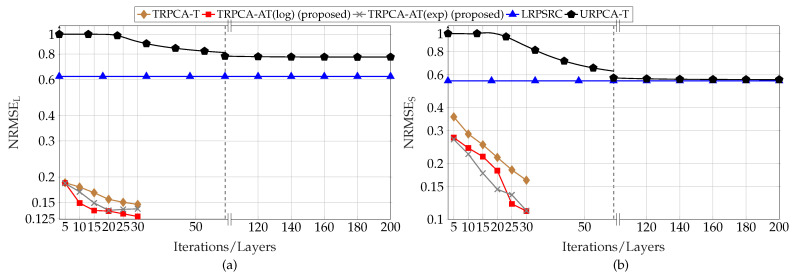
Average NRMSE of low-rank (**a**) and sparsity (**b**) contributions of SFCW Radar model for K/MN=20% with M=N=30 for ADMM-based trained RPCA with thresholding (TRPCA-T), proposed ADMM-based trained RPCA with adaptive thresholding based on logarithm heuristic (TRPCA-AT(log)), proposed ADMM-based trained RPCA with adaptive thresholding based on exponential heuristic (TRPCA-AT(exp)), low-rank plus sparse recovery with convex relaxation (LRPSRC), and ADMM-based untrained RPCA with thresholding (URPCA-T).

Here, we present the results for M=N=30 configuration, i.e., 30 antenna elements and 30 frequency bands. The average normalized RMSEs for the different numbers of layers of the DNN for K/MN=20% is shown in Figure 13. The figure shows that the proposed TRPCA-AT outperforms both URPCA-T and the LRPSRC given in (8). Further, in terms of the average RMSE, the TRPCA-AT and TRPCA-T with five layers outperform URPCA-T with 200 iterations. Therefore, as we compare the number of layers of the TRPCA-AT to the number of iterations of the URPCA-T, the TRPCA-AT achieves a 1:40 improvement for the SFCW radar data, i.e., our proposed approach (TRPCA-AT) is forty times faster than the conventional untrained approach (URPCA-T). Moreover, based on the results shown in Figure 13, the TRPCA-AT shows better performance compared to the TRPCA-T. In addition, note that with 20% compression ratio, the estimation of $Y^{l}$ and s from $y_{cs}$ in (7) is more challenging. Therefore, the average RMSE of the LRPSRC is higher than 0.5. However, the DNN-based TRPCA-AT is able to achieve average RMSE in the range of 0.1 for both sparse and low-rank components. Since $A_{s}$ and $A_{l}$ are unequal in the SFCW radar model, we did not consider the CRB benchmark given in (A4).

**Figure 14:**
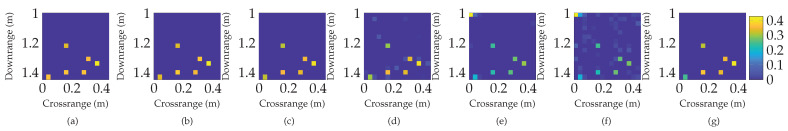
Object recovery for a single case with compression ratio K/MN=20% with M=N=30. (**a**) Ground-truth, (**b**) TRPCA-AT(log) (proposed), (**c**) TRPCA-AT(exp) (proposed), (**d**) TRPCA-T, (**e**) LRPSRC, (**f**) URPCA-T, and (**g**) SP with 100% of data. The proposed TRPCA-AT approaches are able to identify all six objects successfully by only utilizing 20% of data compared to the unweighted approach TRPCA-T.

Next, to further illustrate defect detection, images of the recovered defects are formed, as shown in Figure 14 for a single data sample. As a benchmark, we consider the state-of-the-art subspace projection (SP) [9] method with the full data set, K/MN=100%. Further, for SP, it is assumed that the number of defects is known. Figure 14a shows the actual defect locations. The recovered locations of the defects for the ADMM-based trained RPCA TRPCA-AT(log), TRPCA-AT(exp), TRPCA-T, LRPSRC, ADMM-based untrained RPCA with thresholding URPCA-T, and the SP are shown in Figure 14b–g, respectively. It can be seen that the proposed TRPCA-AT approaches are able to identify all six defects. Further, the proposed TRPCA-AT approaches are even able to estimate amplitudes of the recovered defects (vector s) closer to the actual defects. Therefore, the proposed TRPCA-AT approaches outperform state-of-the-art SP even with 20% of data.

#### 4.2.2. SFCW Large-Scale Simulations

Here, we present the results for M=N=100 configuration, i.e., 100 antenna elements and 100 frequency bands. In small-scale simulations, our proposed approaches TRPCA-AT(log) and TRPCA-AT(exp) achieve similar results, therefore, we chose one of them for the large-scale simulations to compare with the untrained approach URPCA-T.

**Figure 15:**
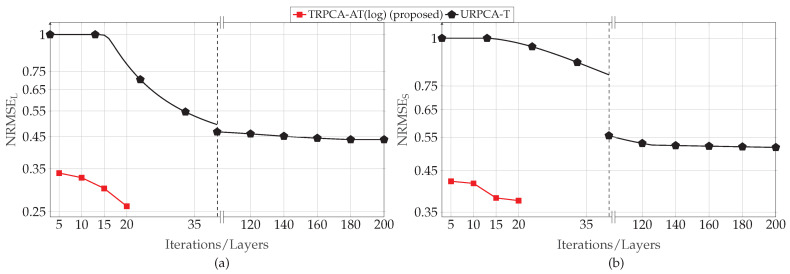
Average NRMSE of low-rank (**a**) and sparsity (**b**) contributions of SFCW Radar model for K/MN=20% with M=N=100 for the proposed ADMM-based trained RPCA with adaptive thresholding based on logarithm heuristic (TRPCA-AT(log)) and ADMM-based untrained RPCA with thresholding (URPCA-T).

The average normalized RMSEs for the different numbers of layers of the DNN for K/MN=20% are shown in Figure 15. The figure shows that the proposed TRPCA-AT(log) outperforms the untrained approach URPCA-T. Further, in terms of the average RMSE, the proposed TRPCA-AT(log) with five layers outperforms the untrained approach URPCA-T with 200 iterations. Therefore, our proposed approach (TRPCA-AT) is forty times faster than the conventional untrained approach (URPCA-T). In addition, note that with 20% compression ratio, the estimations of low-rank and sparse matrices are more challenging. However, the DNN-based TRPCA-AT(log) is able to achieve a lower average NRMSE by parameter tuning compared to the conventional untrained approach (URPCA-T) with the fewer numbers of iterations.

The recovered sparse matrix S contains all the complex reflection coefficients ($\alpha_{p}$) of the defects. Therefore, to further illustrate the defect detectability, we show the total power of the recovered sparse matrix. Here, we consider two metrics: (a) total power of the true locations of the defects and (b) total power of the false detection. Here, the power of the false detection is the power of elements in the sparse matrix S that does not belong to the true locations of the defects. In addition, the total power of the true locations of the defects is the power of elements in the sparse matrix S that belong to the true locations of the defects. These results are shown in Table 4, and it is observed that the proposed approach TRPCA-AT(log) is able to achieve much higher total power of the true locations of the defects than the untrained approach (URPCA-T). Further, it is observed that our approach achieves lower power in false detection, too.

**Figure 16:**
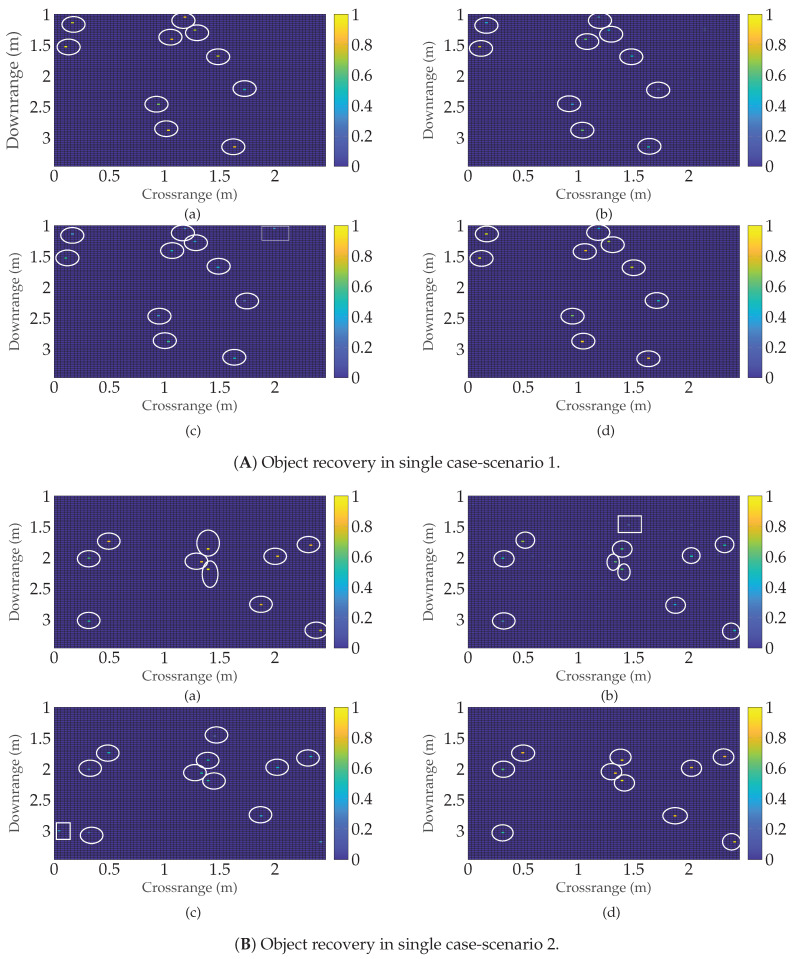
Object recovery for a single case with compression ratio K/MN=20% with M=N=100. (**a**) Ground-truth, (**b**) TRPCA-AT(log) (proposed), (**c**) URPCA-T and (**d**) SP with 100% of data. The proposed TRPCA-AT(log) approach is able to identify all nine objects successfully by only utilizing 20% of data. True locations of the objects are shown inside ellipses and false detections are shown in squares.

Next, to illustrate defect detection, images of the recovered defects are formed for two scenarios as shown in Figure 16. In Figure 16, (Aa) and (Ba) show the actual locations of the defects. The recovered locations of the defects by the proposed ADMM-based trained RPCA TRPCA-AT(log), ADMM-based untrained RPCA with thresholding URPCA-T, and the classical subspace projection (SP) are shown in Figure 16b–d, respectively. It can be seen that the proposed TRPCA-AT(log) approach is able to identify all defects while only utilizing 20% of data. Further, the proposed TRPCA-AT(log) approach has fewer false detections than the untrained RPCA with thresholding (URPCA-T) approach for these two scenarios. It is worth noticing that the conventional SP approach utilizes 100% of data, and for the SP, it is required to know the number of the defects prior.

## 5. Conclusions

This paper presents a deep learning-based parameter tuning for the low-rank plus sparse recovery (RPCA). To this end, an iterative algorithm was developed based on ADMM to estimate the low-rank and sparse contributions with iterative reweighted nuclear and $\ell_{1}$-norm minimization. Next, to improve the accuracies of the recovered low-rank and sparse components and the speed of convergence of the algorithm, we proposed a DNN to tune the parameters of the iterative algorithm, i.e., algorithm unrolling/unfolding. Our proposed approach was evaluated for two types of data. As a standard benchmark, a generic Gaussian data acquisition model was used, and for practical application, the defect detection by SFCW radar from compressive measurements was considered. For both cases, our proposed approach performed substantially better compared to the untrained iterative algorithms in terms of low-rank and sparse recovery and convergence speed. In particular, for compression ratios (K/MN) 50% and 25%, our proposed approach was 15 and 7.5 times faster than the untrained algorithm. In addition to that, we have compared our proposed approach with the state-of-the-art RPCA unfolding approach (CORONA). Our approach achieveed a similar performance level as CORONA for experimental ultrasound imaging data, and our approach outperformed CORONA for generic Gaussian data. Moreover, we analyzed the robustness of our approach for testing signal-to-noise ratio (SNR) uncertainty and the deviation in the measurement matrices ($A_{l}$, $A_{s}$). It was observed that the knowledge of testing SNR is an important factor, and for unknown testing SNR, it is better to train the DNN with SNR like 5 dB. Furthermore, the robust training approach (training with the distribution of deviation) decreased the impact of the deviation in the measurement matrices on the performance. In this work, we considered a model-based unfolding approach where unfolded DNN strictly follows the structure of the optimization steps/rules. As possible future work, it would be interesting to study a model-free unfolding approach which is able to learn new optimization steps/rules from data. Moreover, validation of our approach for experimental/real measurements based on defect detection is subject to future work.

## Figures and Tables

**Table 1:** Comparison of convergence speeds for ADMM-based trained RPCA with thresholding (TRPCA-T), proposed ADMM-based trained RPCA with adaptive thresholding based on logarithm heuristic (TRPCA-AT(log)), proposed ADMM-based trained RPCA with adaptive thresholding based on exponential heuristic (TRPCA-AT(exp)), and ADMM-based untrained RPCA with thresholding (URPCA-T). The proposed approaches TRPCA-AT(log) and TRPCA-AT(exp) are 15 and 7.5 times faster than URPCA-T for compression ratios 50% and 25%, respectively.

Method	Compression Ratio (K/MN) %	Number of Iterations	$\mathrm{NRMSE}=\frac{1}{N_{s}}\sum_{i=1}^{N_{s}}\left({∥X_{i}-{\hat{X}}_{i}∥}_{F}/{∥X_{i}∥}_{F}\right)$	
			Low-Rank Matrix *L*	Sparse Matrix *S*
TRPCA-AT(log)	50%	10	$8.36\times 10^{-2}$	$2.78\times 10^{-2}$
TRPCA-AT(exp)	50%	10	$8.20\times 10^{-2}$	$2.66\times 10^{-2}$
TRPCA-T	50%	10	$8.99\times 10^{-2}$	$3.69\times 10^{-2}$
URPCA-T	50%	150	$9.14\times 10^{-2}$	$4.72\times 10^{-2}$
TRPCA-AT(log)	25%	20	$1.81\times 10^{-1}$	$9.85\times 10^{-2}$
TRPCA-AT(exp)	25%	20	$1.57\times 10^{-1}$	$9.16\times 10^{-2}$
TRPCA-T	25%	20	$2.35\times 10^{-1}$	$1.38\times 10^{-1}$
URPCA-T	25%	150	$2.33\times 10^{-1}$	$1.61\times 10^{-1}$

**Table 2:** Comparison with CORONA [30] for experimental ultrasound imaging data from [30]. CORONA shows slightly better performance compared to the proposed approaches TRPCA-AT(log) and TRPCA-AT(exp) because CORONA is optimized for the structure of the ultrasound data. However, our approaches are not optimized for the structure of the experimental ultrasound data.

Method	Average Recovery Error $=\frac{1}{{\mathrm{MNN}}_{s}}\sum_{i=1}^{N_{s}}\left({∥X_{i}-{\hat{X}}_{i}∥}_{F}\right)$	
	Low-Rank Matrix *L*	Sparse Matrix *S*
CORONA [30]	$3.23\times 10^{-4}$	$3.431\times 10^{-4}$
TRPCA-AT(log)	$3.26\times 10^{-4}$	$6.641\times 10^{-4}$
TRPCA-AT(exp)	$3.37\times 10^{-4}$	$7.101\times 10^{-4}$
TRPCA-T	$9.95\times 10^{-4}$	$7.35\times 10^{-4}$

**Table 3:** Comparison with CORONA [30] for generic Gaussian data. Our proposed approach TRPCA-AT(log) outperforms the CORONA. This is due to the fact that the CORONA is optimized for structured sparsity of ultrasound data, which is not present in generic Gaussian data.

Method	Average Recovery Error $\mathrm{NRMSE}=\frac{1}{N_{s}}\sum_{i=1}^{N_{s}}\left({∥X_{i}-{\hat{X}}_{i}∥}_{F}/{∥X_{i}∥}_{F}\right)$	
	Low-Rank Matrix *L*	Sparse Matrix *S*
CORONA [30]	$4.45\times 10^{-1}$	$4.08\times 10^{-1}$
TRPCA-AT(log)	$6.56\times 10^{-2}$	$3.29\times 10^{-2}$

**Table 4:** Total power of the true defects and false detection for 100 simulations with M=N=100. Here, the total true power of the defects for all simulations is 100.

Method	Total Power $=\sum_{i=1}^{N_{s}}{∥S_{i}∥}_{F}^{2}$	
	True Locations of the Defects	False Detection
URPCA-T	27.8565	2.0247
TRPCA-AT(log)	44.727	1.3537

## Data Availability

The data can be provided by the author U.S.K.P.M.T. upon reasonable request.

## Abbreviations

The following abbreviations and variables are used in this manuscript:

**Abbreviations**	
ADMM	Alternating direction method of multipliers
APG	Accelerated proximal gradient
BS	Background subtraction
CORONA	Convolutional robust principal component analysis
CRB	Cramér–Rao bound
CS	Compressive sensing
DNN	Deep neural network
EM	Electromagnetic
FISTA	Fast iterative soft-thresholding algorithm
GHz	Gigahertz
ISTA	Iterative soft-thresholding algorithm
LISTA	Learned iterative soft-thresholding algorithm
LRPSRC	Low-rank plus sparse recovery with convex relaxation
MSE	Mean squared error
NRMSE	Normalized average root mean squared error
MIMO	Multiple-input and multiple-output
RADAR	Radio detection and ranging
RF	Radio frequency
RPCA	Robust principal component analysis
SFCW	Stepped-frequency continuous wave
SGD	Stochastic gradient descent
SNR	Signal-to-noise ratio
SP	Subspace projection
SVD	Singular value decomposition
TRPCA-AT(exp)	Trained RPCA with adaptive thresholding based on exponential heuristic
TRPCA-AT(log)	Trained RPCA with adaptive thresholding based on logarithm heuristic
TRPCA-T	Trained RPCA with thresholding
URPCA-T	Untrained RPCA with thresholding
**Variable**	
γ∈R	A positive constant used in decay functions
$u\;\in C^{K}$	ADMM auxiliary variables
ρ∈R	ADMM penalty factor
B∈R	Bandwidth of the SFCW radar
$f_{c}\in R$	Carrier frequency of the SFCW radar
$\alpha_{p}\in C$	Complex reflectivity coefficient of the *p*-th defect
$\alpha_{l}\in C$	Complex reflectivity of the layered material structure
$A_{l},\;A_{s},\;A\in C^{K\times MN}$	Compression operators/measurement matrices
K/MN∈R	Compression ratio
$L_{p}\in R$	Learning rate of the parameters ($\lambda_{L}^{t},\gamma^{t},\rho^{t}$) of the DNN
$L_{w}\in R$	Learning rate of the weights ($W_{1},W_{2},W_{3},W_{4}$) of the linear layers of
	the DNN
$L\in C^{M\times N}$	Low-rank matrix
$f_{n}\in R$	n-th frequency band
P∈R	Number of defects
$E_{p}\in R$	Number of epochs
N∈R	Number of frequency bands in SFCW radar system
$N_{s}\in R$	Number of testing samples
$T_{s}\in R$	Number of training samples
M∈R	Number of transceivers in SFCW radar system
Θ	Parameters set that DNN learns ($W_{1},W_{2},W_{3},W_{4}$,$\lambda_{S}^{t}$, $\lambda_{L}^{t},\gamma^{t},\rho^{t}$)
r∈R	Rank of the low-rank matrix L
$Y\in C^{M\times N}$	Received signal matrix corresponding to all *M* transceivers and
	*N* frequencies
$y,\;y_{cs}\in C^{K}$	Reduced received data vector
$Y^{d}\in C^{M\times N}$	Reflection of the defects corresponding to all *M* transceivers and
	*N* frequencies
$Y^{l}\in C^{M\times N}$	Reflection of the layered material structure corresponding to all *M*
	transceivers and *N* frequencies
$\lambda_{l},\;\lambda_{s}\in R$	Regularization parameters
$\Phi \in R^{K\times MN}$	Selection matrix
$\lambda_{L}\in R$	Singular value soft-thresholding parameter
$\sigma \left(L\right)\in \;R^{M}$	Singular values of L
Q∈R	Size of the rectangular grid of the radar scene
$\lambda_{S}\in R$	Soft-thresholding parameter
$S\in C^{M\times N}$	Sparse matrix
$D\in C^{MN\times Q}$	The grid matrix of the radar scene
$s\in C^{Q\times 1}$	Vector that contains all the $\alpha_{p}$ values of the defects
$\lambda_{LT}^{t}\in R^{M}$	Vector that contains all the singular-value threshold values for L in the t+1-th iteration
$\lambda_{ST}^{t}\in R^{MN}$	Vector that contains all the soft-threshold values for S in the t+1-th iteration
$W_{1}^{t}$, $W_{2}^{t}$, $W_{3}^{t}$, $W_{4}^{t}$	Weights of the *t*-th layer of the DNN

## Appendix A

### Appendix A.1. Element-Wise Soft-Thresholding and Singular Value Soft-Thresholding

The element-wise or adaptive soft-thresholding operation is applied to each element of the vector or matrix individually. Here, the main difference of the element-wise or adaptive soft-thresholding compared to the non-adaptive soft-thresholding is that in the element-wise or adaptive soft-thresholding, threshold value is different from one element to another element. However, in non-adaptive or standard soft-thresholding, the same threshold value is applied to all elements. Let us consider a matrix $X\in C^{M\times N}$. To this end, the value after the element-wise or adaptive soft-thresholding $X^{\mathrm{st}}$ is given by

(A1) $$X^{\mathrm{st}}={\mathrm{ST}}_{\lambda_{ST}}\left(X\right).$$

Here, $\lambda_{ST}=\left[\lambda_{ST}^{1,1},\ldots ,\lambda_{ST}^{m,n},\ldots ,\lambda_{ST}^{M,N}\right]$ contains the element-wise thresholds for X. Now, the m-th row and n-th column element of $X^{\mathrm{st}}$ ($x_{m,n}^{\mathrm{st}}$) is given by

(A2) $$x_{m,n}^{\mathrm{st}}={\mathrm{ST}}_{\lambda_{ST}^{m,n}}\left(x_{m,n}\right)=\exp\left(j\theta \right)\;\max\left(|x_{m,n}|-\lambda_{ST}^{m,n},0\right).$$

The m-th row and n-th column element of X is $x_{m,n}$. Further, θ is the phase angle of the $x_{m,n}$ in radians.

In the element-wise or adaptive singular value soft-thresholding, the same concept is applied to the singular values of the matrix as discussed next. The singular value decomposition (SVD) of $X\in C^{M\times N}$ with M≤N is given as $X=U\Lambda V^{H}$. Here, $U\;\in C^{M\times M}$ and $V\;\in C^{N\times N}$ are the matrices of the left and right singular vectors. $\Lambda \;\in \;R^{M\times N}$ is a rectangular diagonal matrix with $\sigma \left(X\right)=\left[\sigma_{1},\ldots \sigma_{m},\ldots ,\sigma_{M}\right]$ on the diagonal and zeros elsewhere. Next, the value after the element-wise or adaptive singular value soft-thresholding $X^{\mathrm{svt}}$ is given by

(A3) $$\begin{array}{cc} X^{\mathrm{svt}} & ={\mathrm{SVT}}_{\lambda_{LT}}\left(X\right)=U\mathrm{diag}\left({\mathrm{ST}}_{\lambda_{LT}}\left(\sigma \left(X\right)\right)\right)V^{H}. \end{array}$$

Note that diag(·) takes a vector and returns the corresponding diagonal matrix. Here, $\lambda_{LT}=\left[\lambda_{LT}^{1},\ldots ,\lambda_{LT}^{m},\ldots \lambda_{LT}^{M}\right]$ contains the different thresholds for each singular value of the X. Next, we briefly discuss the relationship between the adaptive and non-adaptive/non-uniform soft-thresholding. Interestingly, as given in [48], the non-uniform soft-thresholding of a vector $a\;\in R^{M}$ is equivalent to the uniform soft-thresholding (same threshold for all elements) of another vector $b\;\in R^{M}$. Here, $b=\mathrm{sign}\left(a\right)⊙(|a|+w_{0}1_{M}-w)$. $w\;\in R^{M}$ is the non-negative weight vector, and equivalent uniform soft-thresholding value is given by $w_{0}=\max\left(w\right)$. A similar statement is valid for element-wise singular value soft-thresholding, and more detail can be found in [48]. Next, we discuss the computation complexity of the proposed approach.

### Appendix A.2. Computation Complexity

The computational complexity of the proposed DNN is discussed in this subsection. Note that the *t*-th iteration of Algorithm 1 is shown in Figure 2. Here, a single layer of the DNN consists of four dense linear layers, and their weight matrices are given by $W_{1}^{t},\;W_{3}^{t}\in C^{K\times MN}$ and $W_{2}^{t},\;W_{4}^{t}\in C^{MN\times K}$. In the feed-forward propagation, data propagation is given in Equations (19)–(21). Now, for $T_{s}$, number of training samples and $E_{p}$, number of epochs, the computational complexity of the feed-forward propagation is $O(6T_{s}E_{p}\left(MNK\right)+O\left(T_{s}E_{p}\left(M^{2}N+MN^{2}+N^{3}\right)\right)+O\left(T_{s}E_{p}\left(M^{2}N+N^{2}M\right)\right)\;\approx O\left(T_{s}E_{p}\left(MNK+M^{2}N+N^{2}M+N^{3}\right)\right)$. When M=N, the computational complexity of the feed-forward propagation is given by $O(T_{s}E_{p}\left(N^{2}K+N^{3}\right))$. Here, O(·) is the Big O notation for asymptotic computational complexity analysis [49].

For the back propagation, the computational complexity of the linear layers is given by $O(6T_{s}E_{p}\left(MNK\right)$, and for the back propagation through SVD, it is given by $O\left(T_{s}E_{p}\left(M^{2}N+MN^{2}+N^{3}\right)\right)+O\left(T_{s}E_{p}\left(M^{2}N+N^{2}M\right)\right)$. Hence, the training complexity of the DNN is the addition of the feed-forward and the back propagation complexities. Now, for M=N, the training complexity of the DNN is given by $O\left(2T_{s}E_{p}\left(N^{2}K+N^{3}\right)\right)\;\approx O\left(T_{s}E_{p}\left(N^{2}K+N^{3}\right)\right)$. This computational complexity corresponds to the single iteration of the Algorithm 1. Now, for *T* iterations/layers, the training computational complexity is given by $O(TT_{s}E_{p}\left(N^{2}K+N^{3}\right))$. The testing computational complexity is the feed-forward propagation complexity of data through the DNN. It is given by $O(TN_{s}\left(N^{2}K+N^{3}\right))$; here $N_{s}$ is the number of testing samples. Next, for completeness, we discuss the recovery guarantees of the standard RPCA problem in the following.

### Appendix A.3. Cramér–Rao Bound (CRB) and Recovery Guarantees of RPCA

We briefly discuss recovery guarantees of the standard RPCA problem ($A_{l}=A_{s}=I$ in (1)) in this subsection. We consider the above scenario because it is well studied in the literature and well understood, i.e., when the separation of the low-rank matrix L and sparse matrix S is possible. Based on [17], informally, if L is sufficiently low-rank but not sparse and S is sufficiently sparse but not low-rank, the matrices L and S can be estimated exactly with a high probability of success. Here, to solve the RPCA problem, convex relaxations of sparsity and rank in terms of $\ell_{1}$-norm of a matrix and nuclear norm of a matrix as given in (8) is utilized with $\lambda_{l}=1$ and $\lambda_{s}=1/\sqrt{\max(M,N)}$ [17]. Let $\bar{K}=$ max(N,M), $\underline{K}=$ min(N,M) and positive constants $c_{o}$, $p_{s}$ and $p_{r}$. We consider the following theorem from [17].

**Theorem** **A1.**
*It is possible to recover L and S from noiseless observation L+S with a probability at least $1-c_{o}{\bar{K}}^{-10}$ when rank(L)$\;\leq \;p_{r}\underline{K}{\left(\mu \right)}^{-1}{\left(\log\left(\bar{K}\right)\right)}^{-2}$ and ${∥S∥}_{0}\;\leq \;p_{s}\bar{K}\underline{K}$.*

Note that $\bar{K}\underline{K}=MN$. Moreover, μ is an incoherence condition parameter of the low-rank matrix L [17]. As discussed in [56,57,58], when μ is small, the singular value vector of the matrix L is spread out.

Further results are known in terms of the Cramér–Rao bound (CRB) for the RPCA given in [18]. Here, similar to [17], the RPCA problem is solved using the $\ell_{1}$-norm and nuclear norm minimization. Let the received data vector y in (1) follows $y∼N(A\mathrm{vec}\left(L+S\right),\;\sigma^{2}I_{K})$. Here, the matrix $A=A_{l}=A_{s}$ is assumed to be a selection operator which selects a uniformly random subset of size *K* from MN entries. Now, the CRB of unbiased estimation for L and S is bounded by [18]. Since this is the closest matching CRB to our model given in (1), we have considered this formulation as a benchmark:

(A4) $$\begin{array}{c} \left\{s_{0}-N_{0}+\frac{1}{3}\frac{KN_{0}}{K-s_{0}}+\frac{2}{3}\frac{MNN_{0}}{K-s_{0}}\right\}\sigma^{2}\leq \mathrm{CRB}\left(L,S\right)\leq \left\{s_{0}-N_{0}+\frac{3KN_{0}}{K-s_{0}}+\frac{2MNN_{0}}{K-s_{0}}\right\}\sigma^{2}, \end{array}$$

with a probability higher than $1-10\;\exp(-c_{1}/\epsilon_{1}^{2})$, where $∥\mathrm{vec}\left(\hat{L}-L\right){∥}_{2}^{2}+{∥\mathrm{vec}\left(\hat{S}-S\right)∥}_{2}^{2}\leq \epsilon_{1},$ and estimated low-rank and sparse matrices are given by $\hat{L}$ and $\hat{S}$, respectively. Here, $N_{0}=\left(M+N\right)r-r^{2}$ with rank(L)≤r and ${∥S∥}_{0}\leq s_{0}$. As discussed in [18], when M=N and A is an identity matrix, *r* and $s_{0}$ are given by $\mathrm{rank}\left(L\right)\leq \;r=p_{r}N{\left(\log\left(N\right)\right)}^{-5}$$\mathrm{and}\;{∥S∥}_{0}\leq \;s_{0}=p_{s}N^{2}$, respectively.

### Appendix A.4. Three-Stage Training Process for SFCW Data

Here, we describe the three-stage training process that was used to train the DNN for SFCW data. In the first stage, we only learn the $\lambda_{S}^{t}$ and $\lambda_{L}^{t}$ for 50 epochs using Adam. In the second stage, we learn all parameters given in Θ using SGD optimizer for 50 epochs. Finally, we only learn the $\lambda_{S}^{t}$ and $\lambda_{L}^{t}$ for 15 epochs using Adam. In addition, we slightly adjusted the learning rate as the number of layers of the DNN increased. For the first stage, we employed learning rates $1\times 10^{-1}$, $5\times 10^{-2}$, and $5\times 10^{-3}$ for the DNN with 5/10, 15/20, and 25/30 layers, respectively. Here, the only exception is the TRPCA-T. For the TRPCA-T, we considered a learning rate of $5\times 10^{-2}$ for the DNN with 25/30 layers as well. This is due to the fact that the non-adaptive thresholding based TRPCA-T is less sensitive to the change of parameters compared to the adaptive thresholding based TRPCA-AT. For the second and third stages, we employed learning rates $1\times 10^{-3}$, $2.5\times 10^{-4}$ for all layers combinations of the DNN, respectively. The main reason to consider the third training stage is that it achieves higher performance gains with respect to continuation of the second training stage for another 15 epochs. In addition, note that there is a specific reason to use the first stage without directly using the second stage. This is due to the imbalance of $A_{s}$ and $A_{l}$ of the SFCW model compared to the generic Gaussian model. Note that $A_{s}=\Phi D$ and $A_{l}=\Phi$ where Φ is the selection matrix. This matrix has a single non-zero element of value 1 in each row to indicate the selected frequency of a particular antenna if that antenna is selected. However, $A_{s}=\Phi D$ is a combination of the selection matrix and D, where D is generated based on the time delays of the grid (as described in Section 2.1). Therefore, $A_{s}$ has a very specific structure compared to $A_{l}$ and is more difficult to learn. This results in an imbalance in the training phase if we directly start with the stage two, since the NRMSE of the low-rank component tends to be much smaller compared to the sparse component.
